# Economic impact of chicken diseases and other causes of morbidity or mortality in backyard farms in low-income and middle-income countries: a systematic review and meta-analysis

**DOI:** 10.1186/s12917-025-04549-7

**Published:** 2025-03-07

**Authors:** Violeta Muñoz-Gómez, Alexandra P. M. Shaw, Kuban Abdykerimov, Mahmoud Abo-Shehada, Faraz Bulbuli, Duriya Charypkhan, Marina Delphino, Anaïs Léger, Yin Li, Philip Rasmussen, Sukuman Rittem, Bouda Vosough Ahmadi, Paul R. Torgerson

**Affiliations:** 1 Section of Epidemiology, Vetsuisse Faculty, University of Zurich, Winterthurerstrasse 270, Zurich, 8057 Switzerland; 2 Infection Medicine, Biomedical Sciences, Edinburgh Medical School, University of Edinburgh, George Square 1, Edinburgh, EH89JZ UK; 3 Department of Livestock and One Health, Institute of Infection, Veterinary & Ecological Sciences, University of Liverpool, Liverpool, L69 3BX UK; 4 The London School of Hygiene and Tropical Medicine, University of London, Kepple Street, London, EC1E 7HT UK; 5 Lisbon School of Economics and Management, University of Lisbon, Rua do Quelhas 6, Lisbon, 1200-781 Portugal; 6 GenoMar Genetics, Av Madre Leonia Milito, 1500- Gleba Fazenda Palhano, Atsushi Yoshii Tower, Londrina, PR 86050-270 Brazil; 7 Federal Food Safety and Veterinary Office, Schwarzenburgstrasse 155, Bern, 3003 Switzerland; 8 The Commonwealth Scientific and Industrial Research Organisation (CSIRO), 351 Royal Parade, Parkville, Melbourne, VIC 3052 Australia; 9 Department of Veterinary and Animal Sciences, Section for Animal Welfare and Disease Control, University of Copenhagen, Gronnegardsvej 8, Frederiksberg, Copenhagen DK 1870 Denmark; 10 Food and Agriculture Organization of the United Nations (FAO), Viale delle Terme di Caracalla, Rome, 00153 Italy

**Keywords:** Economic, Chicken, Disease, Backyard, Small-holder, Low-income, Middle-income

## Abstract

**Background:**

Backyard chicken farming is usually subsistence and predominates in low-income countries and, to a lesser extent, in middle-income countries. Chicken flocks are generally raised by households in a low-input, low-output system in contact with other flocks, livestock, and wildlife. This low biosecurity setting predisposes chickens to diseases and injuries. A systematic review was conducted to assess the impact of diseases and other causes of mortality in backyard chickens from low income and middle-income countries.

**Results:**

The systematic literature review was conducted following the PRISMA guidelines. Databases consulted included: PubMed, Medline in OVID, Scopus, Web of Knowledge, CAB direct, AGRIS, AgEconSearch, Agricola, Google Scholar, CyberLeninka, CNKI, LILACS, TCI, SID and Civilica. Of the 40,121 studies identified, 78 studies were selected. Only a limited number of studies (*n* = 7) assessed the impact on productivity (weight and egg production losses). Results from the meta-analyses showed that the three main causes of mortality in a production cycle are viral diseases (24.5%, 12.4–42.7), the mix of bacterial and viral diseases (24.2%, 6.2–58.2) and bacterial diseases (11.2%, 4.6–25.0). These three causes of mortality also account for the highest proportion of economic losses for infectious diseases. In the case of non-infectious causes, predation and cachexia are responsible for the highest economic losses in backyard chickens.

**Conclusions:**

Infectious diseases account for the highest economic and mortality losses examined in the selected studies. However, losses due to other causes, such as predation, should not be overlooked. These results could be used to support animal health policy in informing resource allocation to preventive measures to improve food security.

**Supplementary Information:**

The online version contains supplementary material available at 10.1186/s12917-025-04549-7.

## Introduction

The global population reached 8 billion in 2022 [1] and it is predicted to attain the 9 billion by 2037. More than 90% of the population growth from 2022 to 2037 is expected to take place in low and middle-income countries [2] and it is plausible that in these countries, poultry meat will experience an increase in demand for low-cost animal protein driven by this global population growth [3].

In low-income countries, backyard systems represent, on average, 80% of the poultry population [4] and contribute about 98% of poultry products [5]. In Africa, for example, the domestic poultry population raised in village farming exceeds 77% of total poultry production, although this varies between countries [6]. In middle- income countries, the proportion of backyard systems is generally declining due to a shift towards industrial large-scale farms in recent decades [7, 8]. In Thailand, for example, between 1993 and 2013, the share of backyard farmers decreased from 64.2 to 32.1% of total poultry farmers [9]. In China, it has been predicted that the higher integration phase of poultry systems will eventually end backyard poultry systems [8].

Backyard poultry is considered to be subsistence farming and is seen as one of the first steps to tackle issues of malnutrition, food insecurity and poverty [10]. In this production system, chicken products, which are considered a source of high quality animal protein with broad acceptance from religious and cultural background perspectives [10], are mainly used for home consumption [11].

Chicken eggs are a source of protein and micronutrients such as choline and vitamins A, E and B12 [12], , which meet the nutritional needs of adults and children [13]. Egg consumption in children has shown to improve children’s growth and development [12] as well as reducing acute malnutrition [14].

Backyard chickens are generally kept by individual families and often managed by women with support from their children [15]. The sale of birds and eggs generates income to cover basic necessities in the household such as cooking ingredients, clothing, as well as enabling access to education [16] and healthcare [17].

Backyard poultry are raised on a low-input, low-output basis [18–20], with the main input being farmers’ labour. Backyard poultry flocks are normally small, with size varying depending on the region (e.g., 5–20 in Asia, 5–100 in Africa, 10–30 South America) [11] and the farming objectives (e.g., home consumption only, income generation only, and both home consumption and income generation) [11]. Consequently, the size of the flock is constantly changing due to chickens being sold or consumed, eggs hatching or being sold, desynchronisation of egg hatching and chickens dying [11]. Hens in backyard systems can lay between 2.5 and 4 clutches a year, depending on breed and management [11, 21–23] and adult chickens are generally slaughtered between 12 and 20 weeks of age [24], although this might depend on the aim of the farmer (meat production/income generation) [23, 25].

The main factors responsible for low productivity in this production system are low input levels, with sub-optimal management, absence of supplementary feed, presence of diseases, and reduced genetic potential [26]. Local breeds are characterised by slower growth rates, lower laying capacity, and smaller eggs compared to commercial breeds [27, 28]. Although local or native breeds are common in backyard systems, in many cases, birds are crossbred with exotic and/or commercial breeds to increase their production capacity [11]. However, despite having lower productivity, the market prices of meat and eggs from local breeds are between 1.5 and 3 times higher than those of commercial breeds [17] due to consumer preference, which considers them as tastier and of higher quality than commercial breeds (i.e. broiler chickens) [15, 29].

Backyard poultry is characterised by a low dependence on markets for inputs because birds are usually fed with grain, household leftovers, and they scavenge for food (e.g., grass seeds, insects, remains of vegetables and fruit) when outside [7, 11]. Birds in these systems usually have open housing, providing access to an outdoor area during the day [11], and shelter overnight, which allows contact with humans, other birds, livestock, and wildlife [7, 11]. However, in some cases, backyard farms are surrounded by a fence, limiting access to open areas [11, 30] and therefore, contact with potential sources of contamination and predators. Poor biosecurity measures facilitate the entry of pathogens into the chicken flock leading to increased morbidity and premature deaths [4, 7, 11]. Furthermore, since backyard chickens are not regularly monitored for disease, diseases can remain endemic in the flock and in continuous transmission [31]. In addition, water scarcity and poor nutrition also reduce the birds’ productivity and predispose them to be more susceptible to disease and disability. Low biosecurity commercial farms and backyard farms are not often explicitly differentiated by researchers [4]. Both types of farms share characteristics such as selling products in informal markets and a low biosecurity profile [7]. Low-biosecurity commercial farms typically both purchase feed and chicks and sell live birds in various markets that are generally not monitored for health risks [7].

Government- and privately funded interventions to reduce mortality and increase productivity have been carried out in village chicken production systems. These have mainly focused on genetic upgrading, management training, and the provision of infrastructure, farm inputs and services [11, 28]. The impact of visible losses in chickens such as death and low yields has been described in the literature, mainly with case-studies [5]. However, it has never been generalized to backyard chickens in low-income and middle-income countries. The Global Burden of Animal Diseases (GBADs) programme (https://animalhealthmetrics.org/) aims to assess productivity losses and expenditure as a result of diseases in livestock. It acknowledges that small-scale livestock producers, such as backyard farmers, experience economic constraints, and, as part of GBADs, this study contributes to filling that knowledge gap in backyard chickens from low-income and middle-income countries.

## Results

**Fig. 1:**
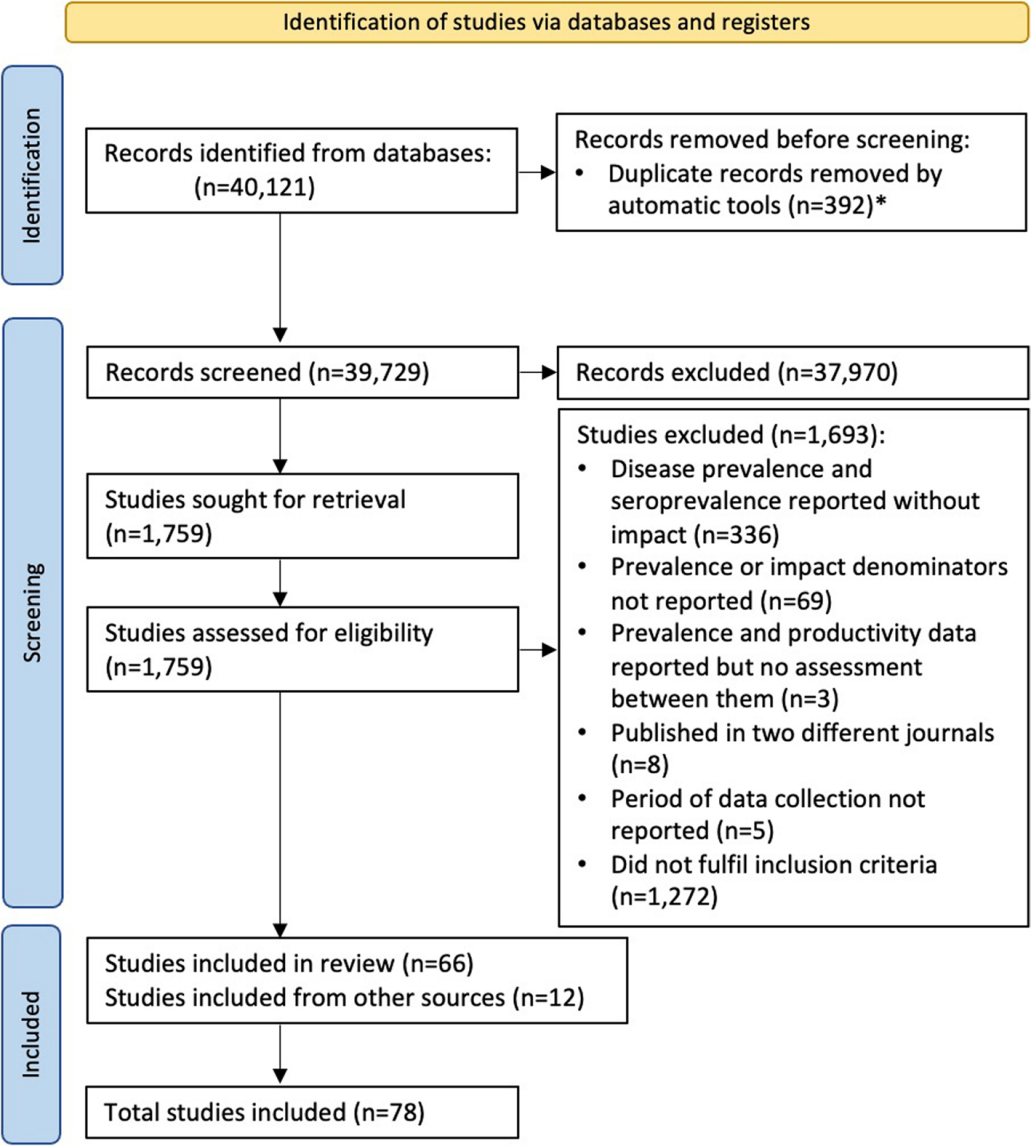
PRISMA flow diagram with search strategy steps. *: The number of duplicates could not be checked on all the platforms and, therefore, they are underestimated

**Fig. 2:**
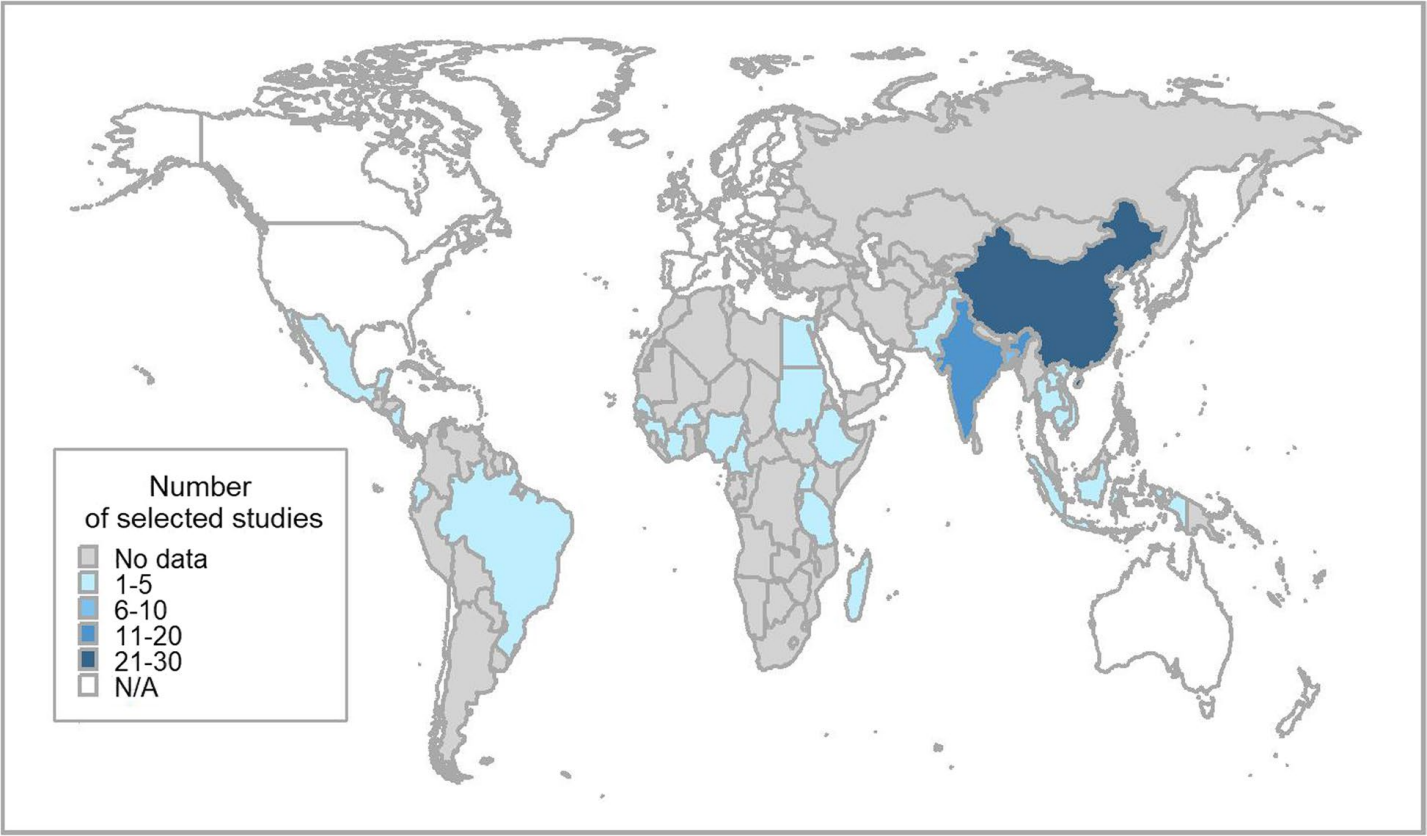
World map showing the geographical distribution where selected studies (n=78) were conducted. The map was generated using the ggplot2 package in R software [32]

The systematic search provided a total of 40,121 articles (Fig. 1). After the screening process, 78 studies were selected. The language of publication of the selected studies was English (*n* = 49), Chinese (*n* = 25), French (*n* = 3), and Thai (*n* = 1). Figure 2 shows the geographical distribution of selected studies (*n* = 78), covering 27 countries.

The results of the risk of bias assessment are available in Additional table S3.

Table 1 provides the descriptive characteristics of the selected studies. Ofthe 78 selected studies, 69 studies (88.5%) reported impacts without a comparator group and 9 studies with one (11.5%) (Table 1). Most studies reported mortality impact (*n* = 71, 91.0%), followed by productivity impact (*n* = 6, 7.7%), and only one study (1.3%) reported both mortality and productivity impacts. Almost none of the studies that were included in this systematic literature review reported data on the secondary outcomes (gross margin and enterprise income) and therefore, these outcomes were not considered in the analysis due to lack of data.

**Table 1:** Descriptive characteristics of selected studies (*n* = 78)

Variable	Number of studies	Frequency (%)
*Study design*		
Studies with a comparator group	9	11.5
Studies without a comparator group	69	88.5
*Type of impact reported*		
Studies that only report mortality impact	71	91.0
Studies that only report productivity impact	6	7.7
Studies that report mortality and productivity impacts	1	1.3
*Type of cause reported*		
Studies that only report infectious cause(s)	50	64.1
Bacteria	5	10.0
Virus	19	38.0
Parasite	18	36.0
Fungus	0	0.0
Mix	8	16.0
Studies that only report non-infectious cause(s)	20	25.6
Injuries/accidents	1	5.0
Predation	11	55.0
Nutritional deficit	1	5.0
Mix (poisoning, predation, injury/accidents, harsh weather)	7	35.0
Studies that report infectious and non-infectious cause (s)	8	10.3
*Number of disease(s)/condition(s) reported*		
Studies that report impact on one disease/condition	53	67.9
Studies that report impact on two disease(s)/condition(s)	12	15.4
Studies that report impact on three disease(s)/condition(s)	7	9.0
Studies that report impact on four or more disease(s)/condition(s)	6	7.7
*Duration of data collection*		
Studies that collect data < 5 months (20 weeks)	44	56.4
Studies that collect data 6–12 months (21–51 weeks)	10	12.8
Studies that collect data ≥ 12 months (≥ 52 weeks)	24	30.8
*Farm data quantified*		
Studies that quantify farm outputs	73	93.6
Studies that quantify farm inputs and outputs	5	6.4
*Number of chickens included in the study*		
≤50	5	6.4
51–200	10	12.8
201–1000	26	33.4
1001–1999	4	5.1
2000–4000	18	23.1
≥4000	14	17.9
NA	1	1.3

*NA *Information not available

Regarding the cause of impact, 50 studies (64.1%) reported impact from infectious causes, 20 studies (25.6%) reported impact from non-infectious causes and 8 studies (10.3%) reported impacts from both infectious and non-infectious causes. Most studies that reported impact from infectious causes targeted viruses (*n* = 19, 38.0%) and parasites (*n* = 18, 36.0%), followed by bacteria (*n* = 5, 10.0%) and a mix of infectious agents (*n* = 8, 16.0%). None of the studies reported only the impact from fungi. In relation to studies that reported the impact from non-infectious causes (*n* = 20, 25.6%), more than half of them reported the impact from predation (*n* = 11, 55.0%), followed by a mix of non-infectious causes (*n* = 7, 35.0%). Only one reported nutritional impact alone (5.0%). Similarly, only one study reported only the impact from injuries/accidents (5.0%). It should be highlighted that, in total, 15 studies, 8 on infectious causes and 7 on non-infectious causes, reported impacts covering different groupscategor of causes.

More than half of the selected studies (67.9%) reported impacts on only one disease/condition, 15.4% on two disease(s)/condition(s), 9.0% on three disease(s)/condition(s) and 7.7% on four or more disease(s)/condition(s). Most of the selected studies reported the impact during a relatively short period of less than or 5 months (*n* = 44, 56.4%) or one year or more (*n* = 24, 30.8%). Only 10 studies covered a period between 6 and 12 months (12.8%). Concerning the quantification of farm data, 73 studies (93.6%) quantified only farm output and only 5 studies (6.4%) reported both, farm outputs and inputs.

Regarding the size of the study (that is, the number of chickens included), we can observe two clusters, less than or equal to 1,000 chickens (*n* = 41, 52.6%) and greater than 1,000 (*n* = 36, 46.2%). Out of the 72 selected studies that reported mortality, 55 were based on endemic scenarios, 16 on epidemic, and one study reported mortality in both circumstances. Tables 4 and 5 display summary information of the studies with comparators for the impact on productivity (weight and loss of egg production) (Table 2) and the impact on mortality (Table 3). In total, 9 studies were found with a comparator out of which, 6 reported only productivity impacts (egg and weight loss), 2 reported only mortality impacts, and 1 reported both mortality and productivity impact. Of the seven studies that reported productivity impact, five focused on parasites, one on nutritional deficiency, and one on a viral disease. Within the five studies that focused on parasites, three of them cover a wide range of parasite species, and two targeted only one parasite species. The weight reductions due to parasites ranged from 4.8 to 41.8% and the losses of egg production were up to 100%. In the case of studies on mortality impacts, three studies targeted viral diseases, one of them in combination with predation. The reduction in mortality in these studies ranged from 3.0 to 82.3%. The level of significance was only reported in four of the seven studies.

**Table 2:** Summary of selected studies with comparators that report productivity impact (weight and egg production losses)

Cause	Pathogen (s)/cause	Number of chickens	Productivity gain		Yield reduction (%)	Reference
			Uninfected or treated chickens	Infected chickens		
Parasite	*Ascaridia galli*, *Heterakis gallinarum*, *cestodes*	68 treated, 65 untreated	65.1 g per week	58.4 g per week	10.3^b^	[33]
Parasite	*A.galli*, *H.gallinarum*, *Raillietina tetragona*,* R. cesticillus*,* R. echonobothrida*,* Amoebotaenia cuneata*,* Capillaria* spp, *Cheilospirura hamulosa*	50 treated, 50 untreated	125.3 g per week	95.8 g per week	23.8^a^	[34]
Parasite	*A.galli*,* H. gallinarum*,* Cheilospirura hamulosa*,* R. tetragona*,* R. cesticillus*,* R. echonobothrida*,* Hymenolepis* spp, *Echinostomatidae*	40 treated, 74 untreated	2090 g total liveweight at the end cycle (~ 18 weeks)	1990 g total liveweight at the end cycle (~ 18 weeks)	4.8^c^	[35]
Parasite	*Eimeria* spp	10 treated, 10 untreated	99.8% weight gain	58% weight gain	41.8 ^c^	[36]
Parasite	*Ascaridia galli*	before (*n* = 800)/ after (*n* = 600)	36.5% hens lay eggs	0% hens lay eggs	100^c^	[37]
Nutritional	Vitamin A deficiency	300 treated, 300 untreated	91 ± 8 g per week	53 ± 17 g per week	41.8^a^	[38]
Virus	Infectious bursal disease virus	1618 vaccinated, 1337 untreated	38.0 g per week	22.0 g per week	42.1^a^	[39]

Level of significance estimated in the study

^a^ statistically significant

^b^non-statistically significant

^c^level of significance not estimated

**Table 3:** Summary of selected studies with comparisons that report the impact of mortality

Cause	Pathogen(s)/ cause	Number of chickens	Mortality/week		Mortality reduction (%)	Reference
			Uninfected or treated chickens	Infected chickens		
Predation and virus	Predation	34 vaccinated, 43 untreated	0.69 (*n* = 34)	0.74 (*n* = 43)	7.2***	[40]
	Newcastle disease virus	10 vaccinated, 52 untreated	0.20 (*n* = 10)	0.89 (*n* = 52)	3.4***	
Virus	Infectious bursal disease virus	1618 vaccinated, 1337 untreated	0.70 (*n* = 114)	2.82 (*n* = 377)	3.0***	[39]
Virus	Avian influenza virus	before (*n* = 3768), after (*n* = 667)	0	1.58 (*n* = 3101)	82.3***	[41]

Level of significance estimated in the study

*** level of significance not estimated; ¤: not reported in the study, assumed to be zero for the calculation

**Fig. 3:**
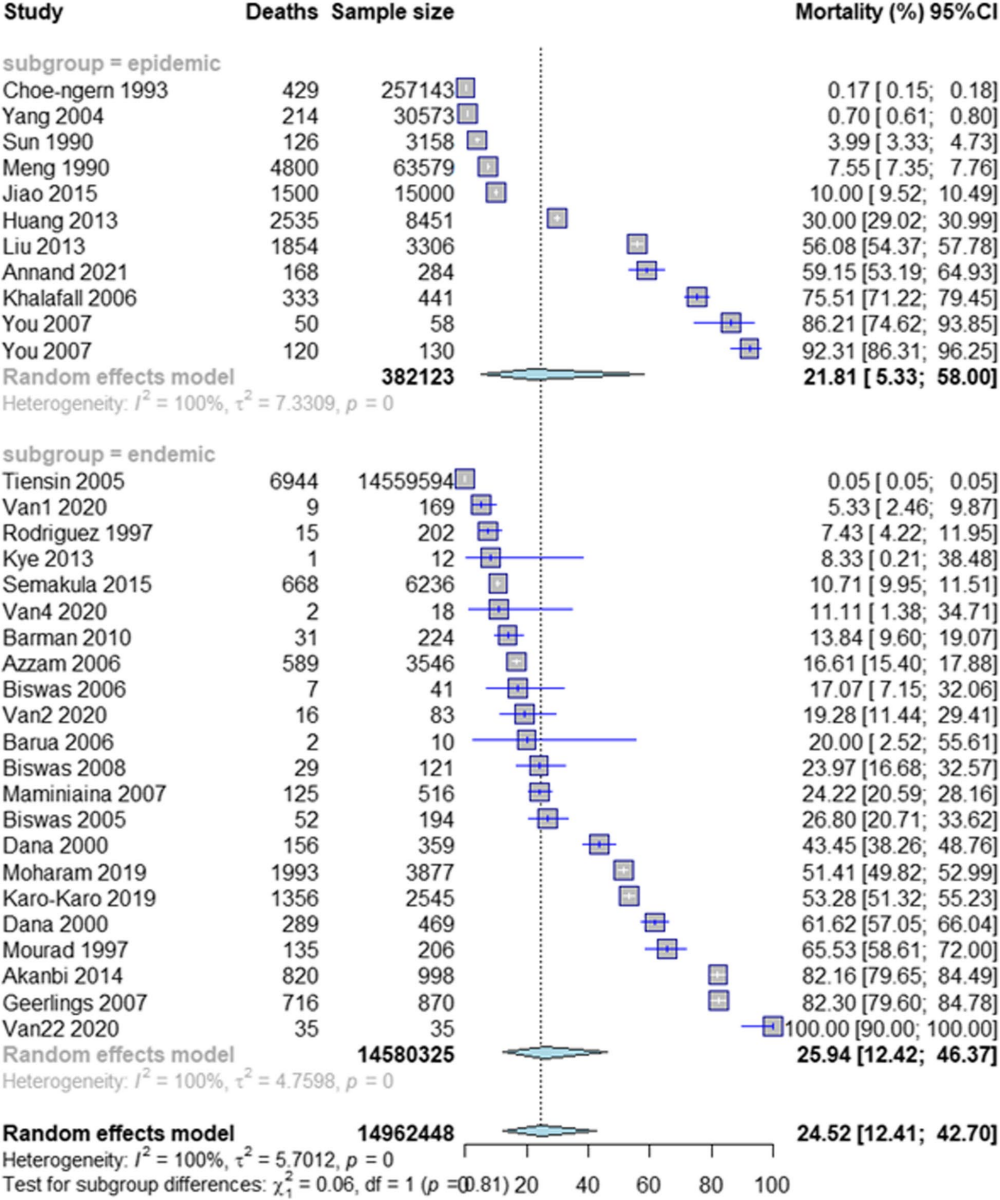
Forest plot of mortality due to viral diseases

The results of the meta-analyses show that, in the selected studies, infectious causes rank higher in mortality than non-infectious causes (Table 4). The five main recorded mortality causes in backyard chicken flocks from low-income and middle-income countries are viral diseases that account for 24.5% (12.4–42.7) of losses per production cycle (Fig. 3), followed by the mix of bacterial and viral diseases with 24.2% (6.8–58.2), bacterial diseases with 11.2% (4.6–25.0), parasitic diseases with 8.5% (5.5–12.9) and predation with 7.8% (4.1–14.3) (Table 4).

**Table 4:** Results of the meta-analyses of mortality impact grouped by infectious and non-infectious

Group of mortality cause	Aetiology	Number of estimates	Pooled mortality per production cycle (%, 95 CI)
*Infectious*			
Viral diseases	Infectious bursal disease virus (IBD), Avian influenza virus (AIV), Avian poxvirus, Chicken anaemia virus, infectious bronchitis disease virus (IB), Avian reovirus, Marek’s disease virus, Reticuloendotheliosis virus, Newcastle disease virus	33	24.5 (12.4–42.7)
Bacterial and viral diseases	*Avibacterium paragallinarum* (AP), IBD, IB, AIV, *Escherichia coli*,* Mycoplasma gallisepticum*,* ornithobacterium rhinotraceale*	16	24.2 (6.8–58.2)
Bacterial diseases	*E. coli*,* Salmonella* spp, *Pasteurella multocida*, AP, *Staphylococcus aureus*,* M. gallisepticum*,* Streptococcus* spp	15	11.2 (4.6–25.0)
Parasitic diseases	*Eimeria* spp, *Histomonas* spp, *Trichomonas* spp, *Ascaridia galli*,* Leucocytozoon* spp, *Raillietina* spp, *Sarcocystiss* spp	21	8.5 (5.5–12.9)
Bacterial and parasitic diseases	*Eimeria* spp *and E. coli*	3	7.6 (2.5–20.9)
Fungal diseases	*Aspergillus* spp, *A. fumigatus*	2	1.5 (0.4–5.1)
*Non-infectious*			
Predation	-	23	7.8 (4.1–14.3)
Cachexia	-	2	7.4 (4.2–12.6)
Weather	-	2	4.8 (3.1–7.4)
Injuries	-	7	2.5 (1.1–5.4)

The sixth and seventh main causes of mortality are the mixture of bacterial and parasitic diseases with 7.6% (2.5–20.9) and cachexia with 7.4% (4.2–12.6). The three lowest causes of mortality, in the selected studies, are fungal infections that account for 1.5% (0.4–5.1) of the losses per production cycle, followed by injuries with 2.5% (1.1–5.4) and poor weather conditions with 4.8% (3.1–7.4). The number of estimates in Table 4 means the number of mortality figures reported for a particular group of mortality causes. Some studies report mortality estimates for more than one group of mortality cause, and that is why the number of mortality estimates for a specific cause sometimes exceeds the number of studies from which they have been extracted.

The mortality causes “sudden death”, “poisoning”, “syndrome” and “multiple causes” were not included in the meta-analyses because each of them had only one mortality estimate and was therefore insufficient to perform a meta-analysis (a minimum of two estimates are required).

The evaluation of potential publication bias influencing the results of the meta-analyses was carried out by examining the asymmetry of the funnel plots and the Peter regression test. For those meta-analyses in which the number of estimates was low (k < 10), the Peter regression test could not be performed and the visual assessment was not considered determinant. As such, the results of the meta-analyses were not adjusted following the trim-and-fill method. In those meta-analyses with enough mortality estimates (k > 10), results of the Peter tests were not significant and, therefore, were not adjusted either. The forest plots, funnel plots and results of the Peter regression tests are presented in Additional files S3 and S4 and additional S4 table, respectively.

The results of the meta-analysis on economic losses (Table 5) indicate that, in general, infectious causes of mortality are responsible for the greatest losses. The five main causes of mortality, which account for the highest proportion of economic losses in backyard chickens from low-income and middle-income countries, are the mix of bacterial and viral diseases (26.7%, 7.5–62.0), viral diseases (25.3%, 13.1–43.3), bacterial diseases (13.9%, 7.8–23.5), parasitic diseases (8.5%, 5.5–12.9), and the mix of bacterial and predation (7.8%, 4.1–14.3). In sixth and seventh place, the mortality causes responsible for the highest economic losses are cachexia, which accounts for 7.7% (5.9–10.1) followed by the mix of bacterial and parasitic diseases with 7.6% (2.5–21.0). At the other end of the spectrum, the three mortality causes that lead to the lowest proportion of the economic value lost are fungal diseases with 1.1% (0.2–4.6), injuries with 2.6% (1.2–5.6) and poor weather conditions with 4.9% (3.7–6.3). The forest plots are available in the Additional S5 file.

**Table 5:** Economic losses from mortality (%) per production cycle grouped by causes

Group of mortality cause	Aetiology	Observed dead chickens ppc^a^	Total sample size ppc^a^	Estimated dead chickens ppc^a, b^	Economic losses (%, 95 CI)
	*Infectious*				
Bacterial and viral diseases	*Avibacterium paragallinarum* (AP), AIV, *Escherichia coli*, IB, IBD, *Mycoplasma gallisepticum*,* Ornithobacterium rhinotraceale*,	125	546	132 (37–318)	26.7 (7.5–62.0)
Viral diseases	Avian influenza virus (AIV), infectious bursal disease virus (IBD), Newcastle disease virus (NDV), Infectious bronchitis virus (IB), Marek’s disease virus (MV), Avian poxvirus, Avian reovirus, chicken anaemia virus	26,118	14,962,447	3,668,792 (1,856,840-6,388,965)	25.3 (13.1–43.3)
Bacterial diseases	AP, *Pasteurella multocida*,* Staphylococcus aureus*,* E. coli*,* Salmonella* spp., *Streptococcus* spp., *M. gallisepticum*	7,722	97,870	10,952 (4,453 − 24,477)	13.9 (7.8–23.5)
Parasitic diseases	*Ascaridia galli*,* Eimeria* spp., *Histomonas* spp., *Trichomonas* spp, *Raillietina* spp., *Leucocytozoon* spp., *Sarcocystiss* spp.	17,535	221,934	18,864 (12,206 − 28,652)	8.5 (5.5–12.9)
Bacterial and parasitic diseases	*E.coli and Eimeria* spp.	2,055	25,953	1,967 (644-5,435)	7.6 (2.5–21.0)
Fungal diseases	*Aspergillus* spp., *A. fumigatus*	4	314	5(1–16)	1.1 (0.2–4.6)
	*Non-infectious*				
Predation	-	3,028	27,767	2,163 (1,138-3,971)	7.8 (4.1–14.3)
Cachexia	-	12	161	12 (7–20)	7.7 (5.9–10.1)
Weather	-	19	397	19 (12–29)	4.9 (3.7–6.3)
Injuries	-	115	4091	101 (49–221)	2.6 (1.2–5.6)

^a ^ppc: per production cycle

^b ^Calculated by multiplying the pooled mortality rate obtained in the meta-analysis by the total sample size per production cycle

The results of the sensitivity analysis (Table 6) show that in the groups of mortality causes in which there are mortality estimates from “epidemic” and “endemic” epidemiological situations, the pooled mortality is higher for Scenario 1 where the assumption is that epidemics occur every year than for Scenario 2 where the assumption is that epidemics occur every two years. In the groups of mortality causes that only contain mortality estimates for an endemic epidemiological situation, namely the mix of bacterial and viral diseases and, fungal diseases, the pooled mortality remains constant in both scenarios.

**Table 6:** Results of the sensitivity analysis of infectious causes considering two scenarios

Mortality cause	Scenario 1: Epidemics occur every year	Scenario 2: Epidemics occur every two years
	Pooled mortality rate (%, 95 CI)	Pooled mortality rate (%, 95 CI)
Viral diseases	14.5 (6.6–29.0)	11.7 (5.3–24.0)
Bacterial and viral diseases	30.5 (8.8–66.7)	30.5 (8.8–66.7)
Parasitic diseases	3.1 (2.0–4.8)	1.9 (1.2–3.3)
Bacterial diseases	7.2 (2.9–16.9)	5.5 (1.9–14.9)
Bacterial and parasitic diseases	1.7 (0.6–5.0)	0.9 (0.2–3.2)
Fungal diseases	1.0 (0.2–4.6)	1.0 (0.2–4.6)

## Discussion

This study suggests that viral diseases are the cause responsible for the highest mortality in backyard chickens in the selected studies, accounting for 24.5% (12.4–42.7) of losses per production cycle, followed by the mix of bacterial and viral diseases with 24.2% (6.8–58.2) and bacterial diseases only with 11.2% (4.6–25.0). This implies that viral diseases, alone or in combination with bacterial diseases, are the leading cause of mortality in backyard chickens per production cycle in the selected studies. Therefore, the implementation of effective prevention and control measures addressing viral diseases can contribute to a reduction in mortality rates. Previous research has also shown that viral diseases, such as Newcastle disease, are the main cause of mortality in backyard chickens in tropical countries, resulting in 100% mortality when virulent strains are present [17]. It should also be noted that for viral diseases, we found more data than for other mortality causes/conditions, and therefore, we should consider whether researchers tend to specifically target viral diseases and examine their impact in backyard chickens. One of the reasons for this could be that the majority of viral pathogens captured in the selected studies cause diseases that are listed by the World Organization for Animal Health (WOAH) [42] and that member countries are required to report [43].

Of the non-infectious causes, predation and cachexia are the main causes of mortality in backyard chickens according to the selected studies, followed by poor weather conditions and injuries. Data for non-infectious causes were extracted as reported by the farmer without further consideration on the aetiology (not reported). We should acknowledge that there might be underlying reasons, such as the presence of comorbidity (i.e., sick chickens have poorer reflexes and less ability to run) or young age (i.e., chicks) that predispose them to fall under these categories. For example, the losses due to cachexia are likely to be related to the presence of an infectious disease and/or a nutritional deficiency, or whereas predation losses are more likely in diseased chickens and chicks. Therefore, we should consider a potential overlap between the mortality losses from infectious and non- infectious causes.

It should be noted that the sum of the pooled mortalities obtained by each group in the meta-analyses exceeds 100%. This is because the pooled mortality was estimated in each group of mortality causes separately, without considering a common ceiling. Rasmussen et al. proposed a model that makes it possible to aggregate the impacts of a range of endemic diseases in livestock without overlap to prevent potential double counting and overestimation of individual disease impacts [44]. Further research will include applying the results of this study in that comorbidity model to account for the productivity and mortality losses attributable to each disease/condition in backyard chickens.

Meta-analyses were performed for each group of mortality cause instead of by specific pathogens, and this influences the interpretation of the results. The pooled mortality obtained for each group assumes that each of the selected studies in that group is a representative sample of the backyard chicken population. However, not all pathogens from each group were examined in each study. For example, for the viral diseases group, some of the studies only report mortality caused by one single pathogen. This is, for example, the case in Tiensin et al. (2005) [45] (Fig. 3), which reported a mortality of 0.05% due to avian influenza in backyard chickens in Thailand.

The pooled mortality obtained for each group of mortality causes should be interpreted as the one that occured in each of the studies for that group if all pathogens belonging to that group were considered. Following the same example, we should interpret that the mortality in the backyard chicken population from Tiensin 2005 would actually be 24.5% if all the viral pathogens of the group had been considered in that population.

We observed that when the selected studies covered an epidemic situation, the data for the analysis were generally gathered over a short period. Mortality estimates from different epidemiological situations (epidemic and endemic) were merged for the meta-analysis under the assumption that when there were data from epidemics, these occured in one production cycle. In the sensitivity analysis, when a group of mortality causes contained data from an epidemic situation, as would be expected, the pooled mortality was lower when the assumption was that epidemics occurred less frequently, however, the magnitude of the effect varied across the different causes. Furthermore, although we only considered infectious causes in the sensitivity analysis, we could expect that in the case of non-infectious causes such as predation, the pooled mortality rate is likely to remain constant throughout the period, unless an underlying health condition or young age predisposes the chickens to that.

In the case of nutritional issues, as stated in the protocol, in the selected studies, these were reported to have been evaluated in the animal by an animal health professional. This requires a physical examination and collection of blood, crop (the anatomical organ), or gizzard samples. However, several studies identified during the screening process assessed the nutritional status of chickens by analysing samples from the crop (the planted field) in which chickens regularly spent time. Therefore, we acknowledge that data collected on nutritional deficiency in backyard chickens in low-income and middle-income countries could be underestimated in this study. However, we concluded that crop field samples were a less reliable proxy for assessing the nutritional status of chickens.

The economic analyses show that infectious causes, namely the mix of bacterial and viral diseases, are responsible for the highest proportion of the value lost in chicken flocks together with viral diseases across the chicken population of the selected studies. Also, the economic losses due to non-infectious causes, such as predation or cachexia, should not be ignored. The mortality causes that account for the highest economic losses usually match those that have the highest pooled mortality rates in the meta-analysis. Interestingly, the estimated dead chickens per production cycle was, in general, higher than the observed dead chickens per production cycle in those groups of mortality causes where there was a great disparity in size among the selected studies. These noticeable differences can be especially observed in the viral diseases group and bacterial diseases group and to a lesser extent in parasitic diseases. These findings show differences in the scope of the study design depending on which pathogens are targeted. Thus, for example, studies of viral diseases were more likely to be conducted at regional/national level than studies of fungal diseases. However, it should also be noted that despite the differences in the sizes of selected studies, the results of the Peter regression tests, where it was conducted, were not significant and, as a result, no further adjustments were necessary in the meta-analyses.

Turning to the price calculations, in economic analysis, the Consumer Price Index (CPI) was selected to estimate the inflation rate in countries because it is based on changes in the prices of a selected “basket” of goods and services which reflects typical consumer expenditure and therefore best matchess the situation of the household for backyard farms. However, it is important to mention that this inflation rate is the average for a basket of goods that are not exclusively livestock products, whose prices might change at a different rate. Although we adjusted the prices to 2020 and therefore, we used CPI data from 2020, these estimates are based on data collected in 2019 [46], meaning that the effect of the Covid 19 pandemic on commodity prices was unlikely to affect this study. The use of the Purchasing Power Parity (PPP) adjusts for the purchasing power differences between countries and thus provides a better comparison between countries. The prices used for the economic analyses were mainly from broilers (commercial breeds) rather than local breeds, although the latter generally have a higher price. The reason for this was the lack of available data. However, we do not think that this affected the results of the economic analyses, as the results were presented as the proportion of the value lost rather than the monetary value.

Mortality is an indicator of animal welfare, environmental impact, and the economic profitability of animal production systems [47]. Resources used in raising animals that end up dying from diseases or other causes of morbidity or mortality are wasted. Furthermore, diseased animals usually have lower productivity and therefore require more natural resources (e.g., feed, water) than healthy animals to achieve the same output, negatively impacting the efficiency of resource use [48]. Therefore, maintaining substantial preventable mortality and morbidity [47] in a production system is neither sustainable nor ethical. In addition, backyard chickens play a role in women’s empowerment, children’s education, and are integrated in cultural events [17, 49]. In middle-income countries, the presence of backyard chickens has been markedly reduced due to vertical integration within the poultry production system [7, 8]. However, we should consider that repressing the backyard production system jeopardizes the socioeconomic benefits and values associated with it [49]. Additionally, raising chickens in a cage rearing system can have costs associated with poor animal welfare such as carcass condemnations, leg problems and increased mortality [50, 51].

More than half of the selected studies only report one disease/condition that meets the inclusion criteria (i.e., infectious diseases and nutritional problems being diagnosed). This finding supports the tendency to target specific diseases. Furthermore, this study only yielded seven articles that looked at the impact of disease on productivity (e.g., weight and egg production losses) and 71 studies on mortality. The lack of studies that look at how disease affects productivity rather than just at mortality is a major gap, which researchers should be encouraged to address. Understanding the effects of disease on the productivity of backyard chickens is essential in order to estimate the economic impact borne by the livestock keeper and what level of expenditure on disease prevention can be justified. In addition, knowing the impacts of the disease can help monitor productivity improvements associated with better animal health over time [52]. The presence of disease in livestock comes with associated expenditures for disease control and prevention [53]. This expenditure has to be added to the productivity and mortality impacts of disease in order to obtain the total the cost of disease at farm level as outlined for the GBADs programme [52].

Although backyard chickens are defined as low-input, low-output production systems and therefore the level of expenditure on conventional treatments (vaccine, drugs) on animal health by farmers is rather low or absent [54], we should considered that the use of traditional treatments (natural remedies) are commonly practiced [55, 56] and that they can reduce the costs of conventional treatment [55].

We could argue that most of the infectious and non-infectious causes that ranked the highest in the mortality and economic losses in this study could be avoided with appropriate management, vaccination, and biosecurity measures. Vaccination against Newcastle disease, for example, has been shown to increase the survival rate in chicks from 30 to 70% in family production systems [11]. However, vaccine costs are sometimes not affordable to farmers and, as such a public-private partnership has been suggested to maintain sustainable vaccination of village poultry [57]. On the other hand, most of the known biosecurity measures have been developed for commercial production systems in middle and high income countries where resources are available and most of the management practices are generally standardized (e.g., sustainable use of disinfectants, access to quality feed, segregation) [4, 58, 59]. However, only a few of these biosecurity measures are suitable and economically viable for backyard systems, and therefore biosecurity measures must be adapted or designed to be applicable in specific circumstances and production capabilities [4, 58]. An example is the use of locally available by-products of agriculture that are not used for human consumption as chicken feed, as alternatives to commercial feeds [59]. Another example is covering the outdoor pens with a net to minimise the contact of chickens with other animals and people external to the household However, the trade-off of reducing contamination and preventing scavenging (cost-free) should be taken into account [59]. An additional example of a low-cost prevention measure against highly pathogenic avian influenza in small-holder chicken producers in Thailand was to raise chickens in separate cycles and clean afterwards. This meant that farmers only raised and sold one clutch at a time rather than raising chickens of various ages throughout the year [60]. Additionally, the participation of farmers through community-led initiatives that encompass a more holistic approach, including empowering women seems promising [54]. An example is a community-based intervention for Newcastle vaccination and biosecurity training in Tanzania. The results showed that the participation of local leaders engaged more people in training and vaccinating a greater number of chickens than otherwise [61].

Looking ahead, the projection of human population growth in low-income and middle-income countries in the next few decades [2] could add additional pressure on backyard chickens, especially in low-income countries where this production system is common [4, 5]. This additional pressure could mean that a higher proportion of households in these countries rely on backyard chicken farming to meet their nutritional and/or economic needs. Therefore, quantifying the main causes of chicken losses in this production system could help prioritize resource allocation in preventive measures to achieve food security.

This study identifies and quantifies the main causes of mortality and economic losses in backyard chickens, and although this assessment was narrowed down to the selected studies from the systematic literature review and therefore these are not global estimates, we covered an extensive number of languages and data sources to identify available published literature. In addition to that, the protocol was rigorous in identifying the aetiology of infectious causes, since as only those studies in which an animal health professional was involved in the health assessment were included.

Language bias was addressed in the search by including the 10 languages spoken mainly in the targeted countries. This approach allowed us to capture more literature than otherwise would have been the case. The complexity in extracting and analysing data following this multilingual approach took more than one year. Therefore, it was not possible to include more updated studies that may have been published since 2022. The fact that 11 people participated in the data selection process could have led to a potential risk of standardisation problems. However, we believe that this challenge was addressed through the provision of clear guidelines, alongside a protocol to standardize the selection of studies and data extraction and regular exchanges and communication within the group.

## Conclusion

In conclusion, infectious causes accounted for the highest economic and mortality losses in the selected studies. However, non-infectious causes such as predation, should not be ignored. Researchers tend to specifically target viral diseases and examine their impact in backyard chickens. The limited number of studies identified that assessed productivity impact (weight and egg production losses) due to diseases highlight the need for more research in this area.

## Methods

### Aims and objectives

The aim of this systematic literature review was to identify and evaluate studies that provided data that could be used to assess the impact of disease and other causes of morbidity or mortality in backyard chickens in low-income and middle-income countries. The following two objectives were formulated following the population (P), exposure (E) and outcome (O) framework [62] (Table 7):

**Table 7:** Components of the objectives according to the PEO framework

Population (*P*)	Chickens living in backyard farms
Exposure (E)/condition of interest	Diseases and other causes of morbidity and/or mortality to chickens
Outcomes (O)	Economic impact and productivity losses

- to assess the economic impact in monetary terms of disease and other causes of morbidity and/or mortality in chicken production of backyard farms in low-income and middle-income countries;
- to assess the productivity losses due to diseases and other causes of morbidity and/or mortality in chicken production at the backyard farm level in low-income and middle-income countries.

### Systematic literature review

This systematic literature review was conducted following the PRISMA guidelines [63] (checklist available in Additional S1 Table).

### Data sources and search strategy

Information sources fall into the category of primary sources (first-hand information) and mainly included journal articles and reports. Main data sources include the following databases: PubMed, Medline in OVID, Scopus, Web of Knowledge, CAB direct, the international information system for agricultural science and technology (AGRIS), research in agricultural and applied economics (AgEconSearch), Agricola, Google Scholar, CyberLeninka, China National Knowledge Infrastructure (CNKI), Literatura Latinoamericana y del Caribe en Ciencias de la Salud (LILACS), ThaiJournal Citation Index (TCI), Scientific Information database (SID), and Civilica. Furthermore, reports and journals from government and international organisations such as the Catalogue de l’École Inter-Etats des Sciences et Médecine Vétérinaires (EISMV) of Dakar, the Revue d’élevage et de médecine vétérinaire des pays tropicaux (REMVT) and International Livestock Research Institute (ILRI) were also included. Only published literature was considered.

The following steps were designed to access scientific publications and grey literature.

1. A basic search of reports and relevant peer-reviewed publications and reports describing backyard chicken farming to identify relevant keywords in English in the title and abstract / foreword was undertaken.
2. The identified keywords/phrases were tested using MEDLINE to ascertain where terms co-occur most, after which they were iteratively refined to improve search terms.
3. The refined search terms were used within specific databases to conduct a comprehensive search of the peer-reviewed literature.
4. Reference lists from selected studies were checked to find additional articles that were not captured in the data search.
5. The records were screened to remove duplicate articles.
6. Partners from the GBADs network were contacted to access relevant unpublished studies.

The search was constrained from 1981 to 2021 (40 years) and eligibility criteria included 10 languages: Arabic, English, Farsi, French, Hindi, Portuguese, Russian, Spanish, Standard Chinese and Thai. The search strategy included: (economic OR productivity OR financial OR expenditure OR control) AND (cost* OR loss* OR impact OR benefit*) AND (chick* OR broiler* OR hen* OR poultry OR “gallus gallus”) AND (disease* OR death* OR mortality OR nutrition*) AND (backyard OR “family-based” OR smallholder OR traditional OR “low biosecurity” OR “subsistence farmer”). However, this search was adapted to the different databases and languages. The search strings applied in each language and in each database together with the date of the search and the number of hits obtained can be found in Additional S1 file.

### Selection criteria

This review covered “low-income” and “middle-income” countries as classified by the World Bank [64]. This review considered all original studies that evaluated the impact of chicken diseases and other causes of morbidity or mortality in backyard farms in selected countries. Studies were accepted or rejected based on three additional criteria: (i) whether the study was about diseases and/or other causes of morbidity or mortality in chickens, (ii) whether the study quantitatively evaluated the relationship between the disease and/or other causes of morbidity or mortality and productivity or economic impact, and (iii) whether the study was conducted in countries included in the previous categories.

This review focused on backyard farms (including free-roaming chicken farms) and low biosecurity commercial farms following the definition by the FAO (sector 3 and 4) [11]. A low level of biosecurity and contact with other birds and wildlife were considered essential selection criteria. Flock size was not considered as a search criterion as this may vary depending on flock dynamics and region. Studies covering all diseases and zoonotic diseases associated with chickens were included. The pathogens identified were cross-checked in the ENHanCEd database of infectious diseases (EID2) (https://eid2.liverpool.ac.uk/). Only studies targeting chickens (*Gallus gallus domesticus*) were included. The “breed” (indigenous or local, commercial, and crossbreed) was not considered as selection criteria because chicken flocks in backyard farms may contain crossbreeds or be referred to using local breed names.

Modelling studies and ex-ante assessment studies were not considered. The targeted study design included experimental studies (clinical field trials) and observational studies (case-controls and longitudinal studies). Only studies in which diseases had been diagnosed by an animal health professional (e.g., veterinarian, para-veterinarian) or confirmed using laboratory diagnosis were included. Similarly, poor nutritional status should have been assessed by an animal health professional. Studies that did not report the period covered in the data collection were excluded.

### Data collection and data extraction

As part of the selection process, the titles were first examined to indicate whether each study contained information on productivity and/or economic impact due to diseases and/or other causes of morbidity or mortality in backyard chickens from low-income and middle-income countries. The full abstract was then assessed independently or in combination with the title in those databases that allowed it, and then, the full text of selected articles was assessed. Methods and risk of bias were assessed using an appraisal tool in the form of a checklist for inclusion in the literature review, which is is available in Additional table S2 and was based on Sargeant et al. (2005) [65]. The purpose of this quality assessment tool was to exclude those studies with a poor quality profile. When ambiguities arose during the screening and assessment process, consensus was reached between at least two of the co-authors (VMG, PR, AS, PT). Data retrieval was carried out by more than one person, and guidelines including a data management plan were provided to ensure consistency between teams in this process. When selected studies included several countries with different income classifications, only data from low-income and middle-income countries were extracted. Similarly, when selected studies included several animal species or zoonotic diseases, only disaggregated data corresponding to chicken species were extracted.

### Outcomes of interest

Primary outcomes include the effect of disease or other causes of morbidity or mortality on productivity (i.e., production losses) and their economic impact in monetary terms at backyard farm level. Secondary outcomes include gross margin and enterprise income. The definition of primary and secondary outcomes can be found in Table 8.

**Table 8:** Definitions of outcomes of interest for the systematic literature review

Primary outcomes
Production parameters: • *Egg production* *Definition*: All eggs produced during the period of reference independently of their utilization (laying, hatching, consumption). This can be expressed as: total weight of eggs/hen, total number of eggs/hens [66]. • *Chicken meat production* *Definition*: Meat produced from the birds on the farm, including meat produced for home consumption, meat sold, meat used to pay for labour, meat given as payment in-kind, meat given as a gift. This could be expressed as: number of birds slaughtered (heads), average live weight/chicken, or total kilograms of live weight [66]. • *Mortality* *Definition*: The number of chickens that died on the farm (excluding culled animals) divided by the total number of chickens present on the farm during the same period and multiplied by 100. This can be expressed as: daily mortality rate or cumulative mortality rate. The mean and standard deviation of the variable were collected, if available. [67] • *Culling rate* *Definition*: Removal of sick/unproductive birds from the flock. Mean and standard deviation of the variable were collected, if available. [18]
Economic impact: *Definition*: It refers to the financial impact at the farm level of a disease or other cause of mortality or morbidity. This includes effects on productivity parameters expressed in monetary terms, expenses associated with disease control and prevention by farmers (e.g. veterinary costs, feed costs, etc.).
Secondary outcomes
• *Gross margin* *Definition*: Computed as the enterprise output less the variable costs that are attributed to it over one year. To calculate the total gross margin of the farm, the gross margins of each enterprise should be summed. The focus was on the gross margin of chickens and it was assumed that the one year enterprise output calculation includes changes in the value of the livestock over that period[68]. • *Enterprise income* Definition: Computed as the enterprise gross margin less the fixed costs attributed to that enterprise. However, it is acknowledged that there might be challenges in attributing fixed costs to individual enterprises [68].

### Meta-analysis of mortality data

Meta-analyses were conducted separately by group of mortality cause, for all groups for which at least two mortality estimates were found. Mortality estimates were classified into groups depending on the aetiology / condition. These groups included “viral diseases”, “fungal diseases”, “bacterial diseases”, “parasitic diseases”, “bacterial and parasitic diseases”, “bacterial and viral diseases”, “predation”, “cachexia”, “weather” and “injuries”. In those groups that are formed by two different etiological groups (e.g. bacterial and parasitic diseases), the same chickens were diagnosed with diseases from both groups.

In addition to this, mortality estimates were grouped as “epidemic” and “endemic” [69] depending on the epidemiological situation described in the study. To conduct the meta-analysis, the number of dead chickens and the number of chickens in the sample size from which the dead chickens arise in each selected study were adjusted for 12 weeks considering the period of the data collection reported in each study. The 12- week period was considered as an approximation of the average length of a production cycle in backyard chickens. This approach allowed us to merge mortality estimates for a production cycle from studies reporting two different epidemiological situations, “epidemic” and “endemic”.

Mortality was interpreted as the incidence of death in a chicken population during a 12-week production cycle. This approach is comparable to the incidence risk (IR = A/B), where “A” are the newly affected chickens in a defined period and “B” the total number of chickens at risk of the condition in that population during that period.

This analysis included mortality data reported in the selected studies with and without a comparator. A comparator group was considered a reference group without the condition/treatment of interest. All analyses were performed in the R software (version 4.1.2, 2022-10-31 ucrt). All meta-analyses were carried out following a random-effects model in logit using the *meta* package [70]. The pooled mortality obtained was then transformed from logit to proportions. When a group of mortality causes had mortality estimates from “epidemic” and “endemic” epidemiological scenarios, the meta-analysis was conducted considering subgroups. Results of the meta-analyses were visualized using forest plots. The asymmetry of the results was assessed using funnel plots and formal statistical tests. Funnel plots were constructed with outcome (mortality, x axis) against the sample size as a measure of variability (y axis) as recommended for the meta-analysis of proportion studies in Hunter et al., 2014 [71]. A Peter regression test, which is based on study size, was conducted to test for asymmetry [71] when there were at least ten estimates for the same group of mortality causes. Forest plots and funnel plots were performed using the *meta* and *metafor* package, respectively [72].

### Sensitivity analysis

The assumption behind the adjustment of mortality estimates for a production cycle from epidemic and endemic situations is that epidemics occur in every production cycle. We conducted a sensitivity analysis for infectious causes by performing two meta-analyses following the methodology as described in meta-analysis in mortality data, in two different scenarios. In the first scenario, we assumed epidemics to occur once a year. For this, the number of chickens in each sample (denominator) from studies categorized as “epidemic” was multiplied by 4.3 (52/12 = 4.3). For the second scenario, we assumed epidemics occurring every two years, and for this, the number of chickens in each sample (denominator) from studies categorized as “epidemic” was multiplied by 8.6 (104/12 = 8.6). For both scenarios, the number of dead chickens (numerator) was taken as reported in each study, regardless of the epidemiological situation. For studies categorized as “endemic”, the number of chickens in the sample was taken as reported in each study.

### Estimation of economic losses

This was undertaken in four stages.

### Literature search of prices

A literature search was conducted to find prices of a broiler chicken and a layer hen at the end of their productive life and day-old-chick (DOC) prices for broilers and layers from countries covered in selected studies. Whenever possible, farm gate prices were used. The year of the price, that is when the research was undertaken, was differentiated from the year of the published reference when this information was available.

### Price conversion in each country

Prices captured in the literature search were all converted to the same year and currency. For this, the inflation rate for each country was estimated using the CPI. The CPI data were obtained from the World Bank (WB) [73]. Prices captured in the literature search were adjusted to 2020 levels, used as a common base and called the ‘final year’, using the formula:

1 $$\:Price\:final\:year=Price\:base\:year*\left(CPI\:finalyear/CPI\:base\:year\right)$$

The year 2020 was identified as the most convenient to adjust because the CPI data were fairly recent and was available for most countries. Once the ‘final year’ price was estimated in the local currency unit, it was converted to US dollars using the currency’s exchange rate for 2020 as given by the WB [74]. When the local price had been reported in a foreign currency (e.g., 1.5 euros for a spent hen in Bangladesh in 2014), the price was converted into the local currency for that year using the WB exchange rate dataset as before [74]. All prices were assumed to apply to the beginning of the year. When prices were captured from 2021, prices were converted to 2020 values using formula [1], and when prices were captured from 2022, they were assumed to be from 2021 (that is,, without inflation). When prices were captured from 2020, the exchange rate in US dollars was directly applied. Further information can be found in Additional file S2.

### Estimation of broiler and layer prices in each country

For each country covered in selected studies, the average prices of a broiler and a layer hen were calculated using the prices that were previously converted. The average price of a broiler chicken was estimated as the mean between the DOC price of a broiler and the price of a broiler chicken at the end of its production life. Similarly, the average price of a layer hen was calculated as the average between the DOC price of a layer hen and the price of a layer hen at the end of its production life. Where possible, the average price of a broiler and a layer was estimated using the DOC price for a chick for the same production purpose. See further details in the Additional files.

Once the average prices of a broiler and a layer hen were calculated for each country, the prices were multiplied by the PPP conversion factor to estimate international prices. The 2020 PPP conversion factor was extracted from WB [75].

The production purpose (meat/eggs/dual) of the chickens of each study was gathered. For those studies in which the production purpose was not stated, a “NA” was assigned. For studies in which the production purpose was “meat” and “eggs”, the average price of a broiler and a layer hen were assigned. For studies in which the production purpose was “dual”, the average price of chickens was estimated as the average between the price of a broiler and the price of a layer hen in that country. In the case of studies where the production purpose was captured as “NA”, the average price of chickens was weighted according to the national production of chickens. This means that if the national chicken population consisted of 70% broilers and 30% layers, the average chicken price for that country was calculated as an appropriately weighted average of the broiler and layer prices.

### Calculation of economic losses

Meta-analyses were conducted for each group of mortality causes to estimate the pooled value lost as a proportion of the monetary value of dead chickens over the monetary value of the sample from which dead chickens arise in each study. The meta-analyses were carried out following the same methodology as described in the meta-analysis of mortality data.

The estimated number of dead chickens was computed by multiplying the pooled mortality rate per production cycle obtained in the meta-analysis of mortality by the total population of chickens in each group of mortality cause per production cycle.

## Supplementary Information

Additional file 1. Summary of searches including search strings, databases, data of search, and number of hits obtained in each of the 10 languages.

Additional file 2. Details of the economic analysis.

Additional file 3. Forest plots of meta-analyses by mortality cause.

Additional file 4. Funnel plots of the meta-analysis by mortality cause.

Additional file 5. Forest plots of the meta-analysis of economic losses by mortality cause.

Additional file 6. References to selected studies for the systematic literature review.

Additional file 7. Dataset used for the analyses.

Additional file 8. PRISMA 2020 checklist.

Additional file 9. Critical appraisal tool.

Additional file 10. Assessment of risk of bias.

Additional file 11. Results of the Peter regression tests.

## Data Availability

The datasets used and/or analysed during the current study are available from the corresponding author on reasonable request.
