# Supplementary material for: Economic impact of chicken diseases and other causes of morbidity or mortality in backyard farms in low-income and middle-income countries: a systematic review and meta-analysis
Source: BMC Vet Res. 2025 Mar 7;21:151. doi: 10.1186/s12917-025-04549-7 (PMC11887245; doi:10.1186/s12917-025-04549-7)
Supplement: Supplementary file 1 — Additional file 1. Summary of searches including search strings, databases, data of search, and number of hits obtained in each of the 10 languages. [file 12917_2025_4549_MOESM1_ESM.docx]

# Summary of searches including search strings, databases, date of search and number of hits obtained in each of the 10 languages

## Arabic language

Table 1. Search string, database, date of search and number of hits obtained in Arabic language

| Database | Search string | Time-frame | Search date | Hits |
| --- | --- | --- | --- | --- |
| Google scholar | مرض AND دجاج | 1981-2021 | 19/10/2021 | 410 |
| Almanhal | دجاج | 1981-2021 | 19/10/2021 | 12 |

## English language

Table 2. Search string, database, date of search and number of hits obtained in English language

| Database | Search string | Time-frame | Search date | Hits |
| --- | --- | --- | --- | --- |
| Pubmed  *additional languages selected: Arabic, Chinese, English, French, Hindi, Persian, Portuguese, Russian, Spanish and Thai | (economic OR productivity OR financial OR expenditure OR control)  AND  (cost*[tiab] OR loss*[tiab] OR impact OR benefit*[tiab])  AND  (chick*[tiab] OR broiler*[tiab] OR hen[tiab] OR hens[tiab] OR poultry OR gallus gallus)  AND  (disease[Mesh] OR disease[tiab] OR death[Mesh] OR death[tiab] OR mortality[Mesh] OR mortalit*[tiab] OR nutrition*[tiab])  AND  (backyard OR family-based OR smallholder OR traditional OR low biosecurity OR subsistence farmer) | 1981-2021 | 23/06/2021 | 256 |
| Medline in OVID | (exp economics/ OR (economic* OR product* OR financial* OR expenditure* OR control*).ti,ab.)  AND (cost* OR loss* OR impact OR benefit*).ti,ab. AND (exp gallus gallus/ OR exp poultry/ OR (chick* OR broiler* OR hen OR hens).ti,ab. adj5  (backyard OR family-based OR smallholder OR traditional OR low biosecurity OR subsistence farmer)) | 1981-2021 | 23/06/2021 | 121 |
| Scopus  *additional languages selected: English, Spanish, French, Chinese, Portuguese, Persian, Russian, Thai | (TITLE-ABS-KEY (economic* OR product* OR financial* OR expenditure* OR control* ) )  AND  (TITLE-ABS-KEY (backyard OR family?based OR smallholder OR traditional OR "low biosecurity" OR “subsistence farmer” W/3 chick* OR broiler* OR hen* OR hens OR poultry OR "gallus gallus" ) ) | 1981-2021 | 10/12/2021 | 1577 |
| Web of Science  *additional language: Russian | TS=(economic*  OR  product*  OR  financial*  OR  expenditure*  OR  control*)   AND TS=(cost* OR loss* OR impact OR benefit*)  AND TI=((backyard  OR  family?based  OR  smallholder  OR  traditional  OR  "low  biosecurity"  OR  subsistence  OR  farmer)  NEAR/5  (chick* OR broiler* OR hen OR hens OR poultry OR "gallus gallus") )  TS: title  TS: topic | 1981-2021 | 30/06/2021 | 207 |
| CAB direct  *additional languages: French, Russian, Hindi, Spanish | (economic OR productivity OR financial OR expenditure OR control) AND (cost* OR loss* OR impact OR benefit*) AND  (chick* OR broiler* OR hen* OR poultry OR “gallus gallus”) AND (disease* OR death* OR mortality OR nutrition*) AND  (backyard OR “family-based” OR smallholder OR traditional OR “low biosecurity” OR “subsistence farmer”)  Chick row and backyard row selected as required to be present in the title. The rest of the rows were selected as to be present in “all fields” (same as Web of Science) | 1981-2021 | 30/06/2021 | 89 |
| AGRIS | (chicken OR broiler OR poultry) AND (backyard OR “family based” OR smallholder OR traditional OR “low biosecurity” OR “subsistence farmer” OR “smallholder”) AND (economic OR productivity) | 1981-2021 | 1/07/2021 | 975 |
| AgEconSearch | 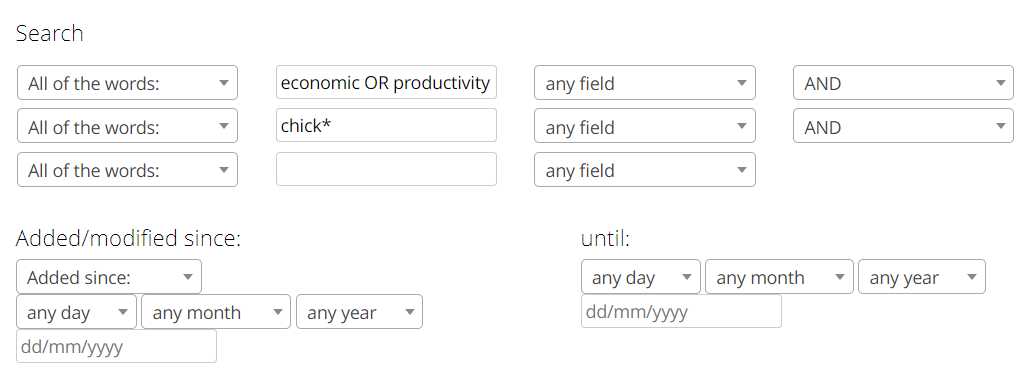 | 1981-2021 | 30/06/2021 | 42 |
| Google scholar | chicken OR broiler AND “economic impact” OR “productivity loss” OR “economic loss” OR “economic cost” OR “productivity impact” OR expenditure AND disease OR mortality OR nutrition AND backyard OR smallholder OR “low biosecurity” | 1981-2021 | 03/02/2022 | 10,600 |
| AGRICOLA | 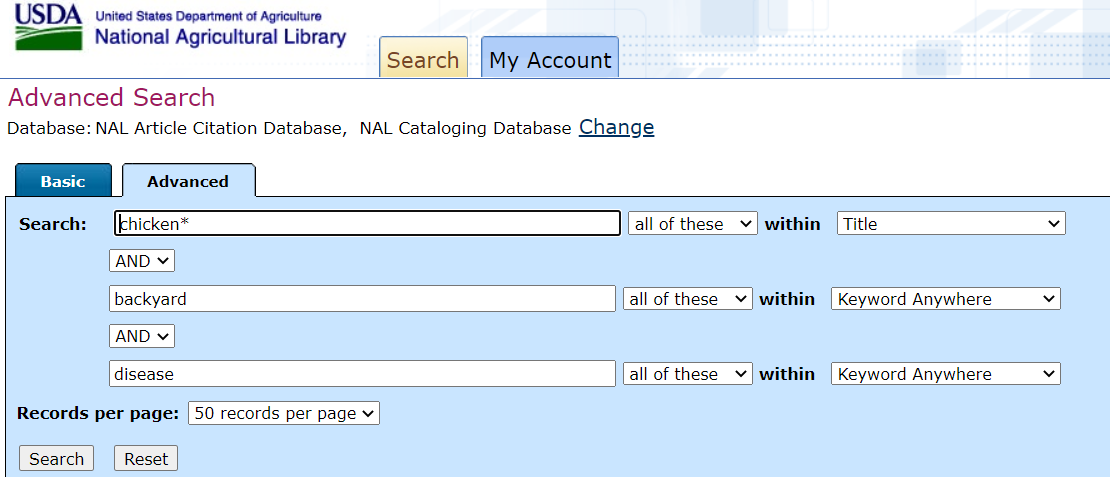 | 1981-2021 | 23/11/2021 | 24 |

## French language

Table 3. Search string, database, date of search and number of hits obtained in French language

| Database | Search string | Time-frame | Search date | Hits |
| --- | --- | --- | --- | --- |
| Google scholar | poulet AND "basse-cour" OR "arrière-cour" AND maladie OR mortalité AND "impact économique" OR "pertes de production" | 1981-2021 | 15/09/2021 | 99 |
| Catalogue de l’Ecole inter-etats des Sciences et médecine vétérinaires de Dakar | poulet et basse-cour | 1981-2021 | 15/09/2021 | 240 |
| Revue d’élevage et de médecine vétérinaire des pays tropicaux (CIRAD) | poulet et basse-cour | 1981-2021 | 15/09/2021 | 7 |
|  | poulet et maladie |  |  | 45 |

## Hindi language

Table 4. Search string, database, date of search and number of hits obtained in Hindi language

| Database | Search string | Time-frame | Search date | Hits |
| --- | --- | --- | --- | --- |
| Google scholar | **मुर्गी AND रोग** | 1981-2021 | 03/02/2022 | 3 |
|  | मुर्गी | 1981-2021 | 03/02/2022 | 12 |

##

## Portuguese language

Table 5. Search string, database, date of search and number of hits obtained in Portuguese language

| Database | Search string | Time-frame | Search date | Hits |
| --- | --- | --- | --- | --- |
| Google scholar | frango AND quintal OR subsistência AND doença OR mortalidade AND “impacto econômico” OR “perdas produtivas” | 1981-2021 | 15/09/2021 | 396 |

## Russian language

Table 6. Search string, database, date of search and number of hits obtained in Russian language

| Database | Search string | Time-frame | Search date | Hits |
| --- | --- | --- | --- | --- |
| Google scholar | бройлер AND домашнее хозяйство | 1981-2021 | 17/09/2021 | 747 |
|  | - "курица" and   болезнь or смерть and экономи* or продуктивность | 1981-2021 | 24/09/2021 | 219 |
|  | болезни домашних птиц | 1981-2021 | 06/12/2021 | 16,200 |
| Cyberleninka | “курица" and болезнь or смерть and эконом* or продуктивность | 1981-2021 | 7/01/2022 | 22 |

## Spanish language

Table 7. Search string, database, date of search and number of hits obtained in Spanish language

| Database | Search string | Time-frame | Search date | Hits |
| --- | --- | --- | --- | --- |
| Google scholar | pollo* OR "aves" OR "gallinas" AND "corral" OR "traspatio" AND "enfermedad" OR "mortalidad" AND "impacto económico" OR "pérdidas productivas" | 1981-2021 | 25/11/2021 | 980 |
| LILACS | Ave$ AND enfermedad$ AND “traspatio” | No option | 25/11/2021 | 5 |

## Standard Chinese language

Table 8. Search string, database, date of search and number of hits obtained in Standard Chinese language

| Database | Search string | Time-frame | Search date | Hits |
| --- | --- | --- | --- | --- |
| Google scholar | 鸡 AND 疾病 AND 后院 OR 小农AND 发表时间 | 1981-2021 | 20/09/2021 | 809 |
| CNKI | 题名=鸡 AND 题名=病 or (Title=中英文扩展(鸡 ) or Title=中英文扩展(病 ) ) AND ( 摘要=损失+危害+影响+死亡率+病死率 AND 摘要=散养+后院+散户+家庭养殖+小规模 ) or ( abstract=中英文扩展(损失)+中英文扩展(危害)+中英文扩展(影响)+中英文扩展(死亡率)+中英文扩展(病死率 ) or abstract=中英文扩展(散养)+中英文扩展(后院)+中英文扩展(散户)+中英文扩展(家庭养殖)+中英文扩展(小规模 ) ) AND 发表时间 | 1981-2021 | 20/09/2021 | 159 |

## Thai language

Table 9. Search string, database, date of search and number of hits obtained in Thai language

| Database | Search string | Time-frame | Search date | Hits |
| --- | --- | --- | --- | --- |
| Google scholar | ไก่ AND โรค AND หลังบ้าน | 1981-2021 | 08/09/2021 | 2,762 |
|  | ไก่ AND โรค AND ฟาร์มเปิด | 1981-2021 | 09/09/2021 | 530 |
|  | สัตว์ปีก AND โรค AND หลังบ้าน | 1981-2021 | 09/09/2021 | 773 |
|  | สัตว์ปีก AND โรค AND ฟาร์มเปิด | 1981-2021 | 09/09/2021 | 200 |
| TCI | ไก่ AND โรค | 1981-2021 | 08/09/2021 | 72 |

## Farsi language

Table 10. Search and results in Civilica in Farsi language

| Type | Name | | Website | | Search date | | Time-frame | Total hits |
| --- | --- | --- | --- | --- | --- | --- | --- | --- |
| Database | Civilica | | <https://civilica.com/> | | 12/12/2021 | | 1981-2021 | 165 |
| Search strings | | **Number of hits** | | **Search strings** | **Number of hits** | **Search strings** | **Number of hits** |  |
| مرغ بومی | | | 102 | خسارات ماکیان بومی | 0 | تلفات جوجه محلی | 0 |  |
| مرغ روستایی | | | 1 | خسارات ماکیان روستایی | 0 | طیور بومی | 23 |  |
| مرغ محلی | | | 3 | خسارات ماکیان محلی | 0 | طیور روستایی | 5 |  |
| مدیریت مرغ بومی | | | 0 | خسارت ماکیان بومی | 0 | طیور محلی | 2 |  |
| مدیریت مرغ روستایی | | | 0 | خسارت ماکیان روستایی | 0 | مدیریت طیور بومی | 0 |  |
| مدیریت مرغ محلی | | | 0 | خسارت ماکیان محلی | 0 | مدیریت طیور روستایی | 1 |  |
| اقتصادی مرغ بومی | | | 4 | تلفات ماکیان بومی | 0 | مدیریت طیور محلی | 0 |  |
| اقتصادی مرغ روستایی | | | 0 | تلفات ماکیان روستایی | 0 | اقتصادی طیور بومی | 0 |  |
| اقتصادی مرغ محلی | | | 0 | تلفات ماکیان محلی | 0 | اقتصادی طیور روستایی | 0 |  |
| خسارات مرغ بومی | | | 0 | جوجه بومی | 20 | اقتصادی طیور محلی | 0 |  |
| خسارات مرغ روستایی | | | 0 | جوجه روستایی | 0 | خسارات طیور بومی |  |  |
| خسارات مرغ محلی | | | 0 | جوجه محلی | 0 | خسارات طیور روستایی | 0 |  |
| خسارت مرغ بومی | | | 0 | مدیریت جوجه بومی | 0 | خسارات طیور محلی | 0 |  |
| خسارت مرغ روستایی | | | 0 | مدیریت جوجه روستایی | 0 | خسارت طیور بومی | 0 |  |
| خسارت مرغ محلی | | | 0 | مدیریت جوجه محلی | 0 | خسارت طیور روستایی | 0 |  |
| تلفات مرغ بومی | | | 0 | اقتصادی جوجه بومی | 0 | خسارت جوجه محلی | 0 |  |
| تلفات مرغ روستایی | | | 0 | اقتصادی جوجه روستایی | 0 | تلفات طیور بومی | 0 |  |
| تلفات مرغ محلی | | | 0 | اقتصادی جوجه محلی | 0 | تلفات طیور روستایی | 0 |  |
| ماکیان بومی | | | 3 | خسارات جوجه بومی | 0 | تلفات طیور محلی | 0 |  |
| ماکیان روستایی | | | 1 | خسارات جوجه روستایی | 0 | اقتصادی ماکیان بومی | 0 |  |
| ماکیان محلی | | | 0 | خسارات جوجه محلی | 0 | اقتصادی ماکیان روستایی | 0 |  |
| مدیریت ماکیان بومی | | | 0 | خسارت جوجه بومی | 0 | اقتصادی ماکیان محلی | 0 |  |
| مدیریت ماکیان روستایی | | | 0 | خسارت جوجه روستایی | 0 | تلفات جوجه روستایی | 0 |  |
| مدیریت ماکیان محلی | | | 0 | تلفات جوجه بومی | 0 |  |  |  |

Table 11. Search and results in the Scientific Information Database (SID) in Farsi language

| Type | Name | Website link | | Search date | | Time-frame | Total hits |
| --- | --- | --- | --- | --- | --- | --- | --- |
| Database | SID | <https://www.sid.ir/> | | 12/12/2021 | | 1981-2021 | 125 |
| Search strings | | **Number of hits** | **Search strings** | **Number of hits** | **Search strings** | **Number of hits** |  |
| مرغ بومی | | 74 | اقتصادی ماکیان محلی | 0 | خسارات جوجه محلی | 0 |  |
| مرغ روستایی | | 0 | خسارات ماکیان بومی | 0 | خسارت جوجه بومی | 0 |  |
| مرغ محلی | | 5 | خسارات ماکیان روستایی | 0 | خسارت جوجه روستایی | 0 |  |
| مدیریت مرغ بومی | | 0 | خسارات ماکیان محلی | 0 | خسارت جوجه محلی | 0 |  |
| مدیریت مرغ روستایی | | 0 | خسارت ماکیان بومی | 0 | تلفات جوجه بومی | 0 |  |
| مدیریت مرغ محلی | | 0 | خسارت ماکیان روستایی | 0 | تلفات جوجه روستایی | 0 |  |
| اقتصادی مرغ بومی | | 0 | خسارت ماکیان محلی | 0 | تلفات جوجه محلی | 0 |  |
| اقتصادی مرغ روستایی | | 0 | تلفات ماکیان بومی | 0 | طیور بومی | 18 |  |
| اقتصادی مرغ محلی | | 0 | تلفات ماکیان روستایی | 0 | طیور روستایی | 2 |  |
| خسارات مرغ بومی | | 0 | تلفات ماکیان محلی | 0 | طیور محلی | 1 |  |
| خسارات مرغ روستایی | | 0 | جوجه بومی | 11 | مدیریت طیور بومی | 0 |  |
| خسارات مرغ محلی | | 0 | جوجه روستایی | 0 | مدیریت طیور روستایی | 0 |  |
| خسارت مرغ بومی | | 0 | جوجه محلی | 2 | مدیریت طیور محلی | 0 |  |
| خسارت مرغ روستایی | | 0 | مدیریت جوجه بومی | 0 | اقتصادی طیور بومی | 0 |  |
| خسارت مرغ محلی | | 0 | مدیریت جوجه روستایی | 0 | اقتصادی طیور روستایی | 0 |  |
| تلفات مرغ بومی | | 0 | مدیریت جوجه محلی | 0 | اقتصادی طیور محلی | 0 |  |
| تلفات مرغ روستایی | | 0 | اقتصادی جوجه بومی | 0 | خسارات طیور بومی | 0 |  |
| تلفات مرغ محلی | | 0 | اقتصادی جوجه روستایی | 0 | خسارات طیور روستایی | 0 |  |
| ماکیان بومی | | 12 | اقتصادی جوجه محلی | 0 | خسارات طیور محلی | 0 |  |
| ماکیان روستایی | | 0 | خسارات جوجه بومی | 0 | خسارت طیور بومی | 0 |  |
| ماکیان محلی | | 0 | خسارات جوجه روستایی | 0 | خسارت طیور روستایی | 0 |  |
| مدیریت ماکیان بومی | | 0 | تلفات طیور محلی | 0 | خسارت طیور محلی | 0 |  |
| مدیریت ماکیان روستایی | | 0 | اقتصادی ماکیان روستایی | 0 |  | |  |
| مدیریت ماکیان محلی | | 0 | تلفات طیور روستایی | 0 |  |  |  |
| اقتصادی ماکیان بومی | | 0 | تلفات طیور بومی | 0 |  |  |  |

Table 12-Search and results in Iran Journal in Farsi language

| Type | Name | Website link | | Search date | | Time-frame | Total hits |
| --- | --- | --- | --- | --- | --- | --- | --- |
| Journal | Iran Journal | <https://iranjournals.nlai.ir/> | | 12/12/2021 | | 1981-2021 | 1181 |
| Search strings | | **Number of hits** | **Search strings** | **Number of hits** | **Search strings** | **Number of hits** |  |
| مرغ بومی | | 208 | اقتصادی ماکیان بومی | 10 | خسارت جوجه بومی | 3 |  |
| مرغ روستایی | | 82 | اقتصادی ماکیان روستایی | 3 | خسارت جوجه روستایی | 0 |  |
| مرغ محلی | | 22 | اقتصادی ماکیان محلی | 0 | خسارت جوجه محلی | 0 |  |
| مدیریت مرغ بومی | | 9 | خسارات ماکیان بومی | 5 | تلفات جوجه بومی | 11 |  |
| مدیریت مرغ روستایی | | 21 | خسارات ماکیان روستایی | 0 | تلفات جوجه روستایی | 0 |  |
| مدیریت مرغ محلی | | 2 | خسارات ماکیان محلی | 0 | تلفات جوجه محلی | 4 |  |
| اقتصادی مرغ بومی | | 38 | خسارت ماکیان بومی | 1 | طیور بومی | 188 |  |
| اقتصادی مرغ روستایی | | 19 | خسارت ماکیان روستایی | 0 | طیور روستایی | 69 |  |
| اقتصادی مرغ محلی | | 4 | خسارت ماکیان روستایی | 1 | طیور محلی | 25 |  |
| خسارات مرغ بومی | | 5 | تلفات ماکیان بومی | 3 | مدیریت طیور بومی | 17 |  |
| خسارات مرغ روستایی | | 0 | تلفات ماکیان روستایی | 1 | مدیریت طیور روستایی | 19 |  |
| خسارات مرغ محلی | | 1 | تلفات ماکیان محلی | 0 | مدیریت طیور محلی | 6 |  |
| خسارت مرغ بومی | | 2 | جوجه بومی | 93 | اقتصادی طیور بومی | 41 |  |
| خسارت مرغ روستایی | | 13 | جوجه روستایی | 38 | اقتصادی طیور روستایی | 27 |  |
| خسارت مرغ محلی | | 0 | جوجه محلی | 10 | اقتصادی طیور محلی | 7 |  |
| تلفات مرغ بومی | | 13 | مدیریت جوجه بومی | 8 | خسارات طیور بومی | 12 |  |
| تلفات مرغ روستایی | | 6 | مدیریت جوجه روستایی | 7 | خسارات طیور بومی | 2 |  |
| تلفات مرغ محلی | | 3 | مدیریت جوجه محلی | 2 | خسارات طیور محلی | 2 |  |
| ماکیان بومی | | 37 | اقتصادی جوجه بومی | 17 | خسارت طیور بومی | 3 |  |
| ماکیان روستایی | | 8 | اقتصادی جوجه روستایی | 4 | خسارت طیور روستایی | 1 |  |
| ماکیان محلی | | 3 | اقتصادی جوجه محلی | 2 | خسارت طیور محلی | 1 |  |
| مدیریت ماکیان بومی | | 5 | خسارات جوجه بومی | 5 | تلفات طیور بومی | 14 |  |
| مدیریت ماکیان روستایی | | 4 | خسارات جوجه روستایی | 0 | تلفات طیور روستایی | 8 |  |
| مدیریت ماکیان محلی | | 1 | خسارات جوجه روستایی | 1 | تلفات طیور محلی | 4 |  |

Table 13- Search and results in Veterinary Researches and Biological Products (VJ) / مجله تحقیقات دامپزشکی و فرآورده‌های بیولوژیک

| Type | Name | Website link | | Search date | | Time-frame | Total hits |
| --- | --- | --- | --- | --- | --- | --- | --- |
| Journal | VJ | <https://vj.areeo.ac.ir/> | | 12/12/2021 | | 1988^[[1]](#footnote-1)^-2021 | 6 |
| Search strings | | **Number of hits** | **Search strings** | **Number of hits** | **Search strings** | **Number of hits** |  |
| مرغ بومی | | 4 | مدیریت ماکیان روستایی | 0 | اقتصادی جوجه محلی | 0 |  |
| مرغ روستایی | | 0 | مدیریت ماکیان محلی | 0 | خسارات جوجه بومی | 0 |  |
| مرغ محلی | | 0 | اقتصادی ماکیان بومی | 0 | خسارات جوجه روستایی | 0 |  |
| مدیریت مرغ بومی | | 0 | اقتصادی ماکیان روستایی | 0 | خسارات جوجه محلی | 0 |  |
| مدیریت مرغ روستایی | | 0 | اقتصادی ماکیان محلی | 0 | خسارت جوجه بومی | 0 |  |
| مدیریت مرغ محلی | | 0 | خسارات ماکیان بومی | 0 | خسارت جوجه روستایی | 0 |  |
| اقتصادی مرغ بومی | | 0 | خسارات ماکیان روستایی | 0 | خسارت جوجه محلی | 0 |  |
| اقتصادی مرغ روستایی | | 0 | خسارات ماکیان محلی | 0 | تلفات جوجه بومی | 0 |  |
| اقتصادی مرغ محلی | | 0 | خسارت ماکیان بومی | 0 | تلفات جوجه روستایی | 0 |  |
| خسارات مرغ بومی | | 0 | خسارت ماکیان روستایی | 0 | تلفات جوجه محلی | 0 |  |
| خسارات مرغ روستایی | | 0 | خسارت ماکیان محلی | 0 | طیور بومی | 1 |  |
| خسارات مرغ محلی | | 0 | تلفات ماکیان بومی | 0 | طیور روستایی | 0 |  |
| خسارت مرغ بومی | | 0 | تلفات ماکیان روستایی | 0 | طیور محلی | 0 |  |
| خسارت مرغ روستایی | | 0 | تلفات ماکیان محلی | 0 | مدیریت طیور بومی | 0 |  |
| خسارت مرغ محلی | | 0 | جوجه بومی | 0 | مدیریت طیور روستایی | 0 |  |
| تلفات مرغ بومی | | 0 | جوجه روستایی | 0 | مدیریت طیور محلی | 0 |  |
| تلفات مرغ روستایی | | 0 | جوجه محلی | 0 | اقتصادی طیور بومی | 0 |  |
| تلفات مرغ محلی | | 0 | مدیریت جوجه بومی | 0 | اقتصادی طیور روستایی | 0 |  |
| ماکیان بومی | | 1 | مدیریت جوجه روستایی | 0 | اقتصادی طیور محلی | 0 |  |
| ماکیان روستایی | | 0 | مدیریت جوجه محلی | 0 | خسارات طیور بومی | 0 |  |
| ماکیان محلی | | 0 | اقتصادی جوجه بومی | 0 | خسارات طیور روستایی | 0 |  |
| ماکیان محلی | | 0 | اقتصادی جوجه روستایی | 0 | خسارات طیور محلی | 0 |  |
| خسارت طیور بومی | | 0 | خسارت طیور روستایی | 0 | خسارت طیور محلی | 0 |  |
| تلفات طیور بومی | | 0 | تلفات طیور روستایی | 0 | تلفات طیور روستایی | 0 |  |

Table 14- Search and results in Ferdowsi University of Mashhad(Um: Profdoc) in Farsi language

| Type | Name | Website link | | Search date | | Time-frame | Total hits |
| --- | --- | --- | --- | --- | --- | --- | --- |
| Journal | Profdoc | <https://profdoc.um.ac.ir/> | | 12/12/2021 | | 1981-2021 | 8 |
| Search strings | | **Number of hits** | **Search strings** | **Number of hits** | **Search strings** | **Number of hits** |  |
| مرغ بومی | | 5 | مدیریت ماکیان بومی | 0 | اقتصادی جوجه بومی | 0 |  |
| مرغ روستایی | | 0 | مدیریت ماکیان روستایی | 0 | اقتصادی جوجه روستایی | 0 |  |
| مرغ محلی | | 0 | مدیریت ماکیان محلی | 0 | اقتصادی جوجه محلی | 0 |  |
| مدیریت مرغ بومی | | 0 | اقتصادی ماکیان بومی | 0 | اقتصادی جوجه محلی | 0 |  |
| مدیریت مرغ روستایی | | 0 | اقتصادی ماکیان روستایی | 0 | خسارات جوجه بومی | 0 |  |
| مدیریت مرغ محلی | | 0 | اقتصادی ماکیان محلی | 0 | خسارات جوجه روستایی | 0 |  |
| اقتصادی مرغ بومی | | 0 | خسارات ماکیان بومی | 0 | خسارات جوجه محلی | 0 |  |
| اقتصادی مرغ روستایی | | 0 | خسارات ماکیان روستایی | 0 | خسارت جوجه بومی | 0 |  |
| اقتصادی مرغ محلی | | 0 | خسارات ماکیان محلی | 0 | خسارت جوجه روستایی | 0 |  |
| خسارات مرغ بومی | | 0 | خسارت ماکیان بومی | 0 | خسارت جوجه محلی | 0 |  |
| خسارات مرغ روستایی | | 0 | خسارت ماکیان روستایی | 0 | تلفات جوجه بومی | 0 |  |
| خسارات مرغ محلی | | 0 | خسارت ماکیان محلی | 0 | تلفات جوجه روستایی | 0 |  |
| خسارت مرغ بومی | | 0 | تلفات ماکیان بومی | 0 | تلفات جوجه محلی | 0 |  |
| خسارت مرغ روستایی | | 0 | تلفات ماکیان روستایی | 0 | طیور بومی | 3 |  |
| خسارت مرغ محلی | | 0 | تلفات ماکیان محلی | 0 | طیور روستایی | 0 |  |
| تلفات مرغ بومی | | 0 | جوجه بومی | 0 | طیور محلی | 0 |  |
| تلفات مرغ روستایی | | 0 | جوجه روستایی | 0 | مدیریت طیور بومی | 0 |  |
| تلفات مرغ محلی | | 0 | جوجه محلی | 0 | مدیریت طیور روستایی | 0 |  |
| ماکیان بومی | | 0 | مدیریت جوجه بومی | 0 | مدیریت طیور محلی | 0 |  |
| ماکیان روستایی | | 0 | مدیریت جوجه روستایی | 0 | اقتصادی طیور بومی | 0 |  |
| ماکیان محلی | | 0 | مدیریت جوجه محلی | 0 | اقتصادی طیور روستایی | 0 |  |
| اقتصادی طیور محلی | | 0 | خسارات طیور بومی | 0 | خسارات طیور روستایی | 0 |  |
| خسارات طیور محلی | | 0 | خسارت طیور بومی | 0 | خسارت طیور روستایی | 0 |  |
| خسارت طیور محلی | | 0 | تلفات طیور بومی | 0 |  |  |  |
| تلفات طیور محلی | | 0 | تلفات طیور روستایی | 0 |  |  |  |

Table 15-Search and results in the Journal of Veterinary Research (JVR) /مجله تحقیقات دامپزشکی

| Type | Name | Website link | | Search date | | Time-frame | Total hits |
| --- | --- | --- | --- | --- | --- | --- | --- |
| Journal | JVR | <https://jvr.ut.ac.ir/> | | 12/12/2021 | | 1981-2021 | 7 |
| Search strings | | **Number of hits** | **Search strings** | **Number of hits** | **Search strings** | **Number of hits** |  |
| مرغ بومی | | 0 | اقتصادی ماکیان بومی | 0 | خسارت جوجه بومی | 0 |  |
| مرغ روستایی | | 0 | اقتصادی ماکیان روستایی | 0 | خسارت جوجه روستایی | 0 |  |
| مرغ محلی | | 0 | اقتصادی ماکیان محلی | 0 | خسارت جوجه محلی | 0 |  |
| مدیریت مرغ بومی | | 0 | خسارات ماکیان بومی | 0 | تلفات جوجه بومی | 0 |  |
| مدیریت مرغ روستایی | | 0 | خسارات ماکیان روستایی | 0 | تلفات جوجه روستایی | 0 |  |
| مدیریت مرغ محلی | | 0 | خسارات ماکیان محلی | 0 | تلفات جوجه محلی | 0 |  |
| اقتصادی مرغ بومی | | 0 | خسارت ماکیان بومی | 0 | طیور بومی | 4 |  |
| اقتصادی مرغ روستایی | | 0 | خسارت ماکیان روستایی | 0 | طیور روستایی | 2 |  |
| اقتصادی مرغ محلی | | 0 | خسارت ماکیان محلی | 0 | طیور محلی | 0 |  |
| خسارات مرغ بومی | | 0 | تلفات ماکیان بومی | 0 | مدیریت طیور بومی | 0 |  |
| خسارات مرغ روستایی | | 0 | تلفات ماکیان روستایی | 0 | مدیریت طیور روستایی | 0 |  |
| خسارات مرغ محلی | | 0 | تلفات ماکیان محلی | 0 | مدیریت طیور محلی | 0 |  |
| خسارت مرغ بومی | | 0 | جوجه بومی | 0 | اقتصادی طیور بومی | 0 |  |
| خسارت مرغ روستایی | | 0 | جوجه روستایی | 0 | اقتصادی طیور روستایی | 0 |  |
| خسارت مرغ محلی | | 0 | جوجه محلی | 0 | اقتصادی طیور محلی | 0 |  |
| تلفات مرغ بومی | | 0 | مدیریت جوجه بومی | 0 | خسارات طیور بومی | 0 |  |
| تلفات مرغ روستایی | | 0 | مدیریت جوجه روستایی | 0 | خسارات طیور روستایی | 0 |  |
| تلفات مرغ محلی | | 0 | مدیریت جوجه محلی | 0 | خسارات طیور محلی | 0 |  |
| ماکیان بومی | | 1 | اقتصادی جوجه بومی | 0 | خسارت طیور بومی | 0 |  |
| ماکیان روستایی | | 0 | اقتصادی جوجه روستایی | 0 | خسارت طیور روستایی | 0 |  |
| ماکیان محلی | | 0 | اقتصادی جوجه محلی | 0 | خسارت طیور محلی | 0 |  |
| مدیریت ماکیان بومی | | 0 | خسارات جوجه بومی | 0 | تلفات طیور بومی | 0 |  |
| مدیریت ماکیان روستایی | | 0 | خسارات جوجه روستایی | 0 | تلفات طیور روستایی | 0 |  |
| مدیریت ماکیان محلی | | 0 | خسارات جوجه محلی | 0 | تلفات طیور محلی | 0 |  |

Table 16-Search and results in Journal of Livestock Research (JLR) /مجله تحقیقات دام و طیور

| Type | Name | Website link | | Search date | | Time-frame | Total hits |
| --- | --- | --- | --- | --- | --- | --- | --- |
| Journal | JLR | <https://jlr.birjand.ac.ir/> | | 12/12/2021 | | 2012^[[2]](#footnote-2)^-2021 | 4 |
| Search strings | | **Number of hits** | **Search strings** | **Number of hits** | **Search strings** | **Number of hits** |  |
| مرغ بومی | | 4 | مدیریت ماکیان بومی | 0 | اقتصادی جوجه بومی | 0 |  |
| مرغ روستایی | | 0 | مدیریت ماکیان روستایی | 0 | اقتصادی جوجه روستایی | 0 |  |
| مرغ محلی | | 0 | مدیریت ماکیان محلی | 0 | اقتصادی جوجه محلی | 0 |  |
| مدیریت مرغ بومی | | 0 | اقتصادی ماکیان بومی | 0 | خسارات جوجه بومی | 0 |  |
| مدیریت مرغ روستایی | | 0 | اقتصادی ماکیان روستایی | 0 | خسارات جوجه روستایی | 0 |  |
| مدیریت مرغ محلی | | 0 | اقتصادی ماکیان محلی | 0 | خسارات جوجه محلی | 0 |  |
| اقتصادی مرغ بومی | | 0 | خسارات ماکیان بومی | 0 | خسارت جوجه بومی | 0 |  |
| اقتصادی مرغ روستایی | | 0 | خسارات ماکیان روستایی | 0 | خسارت جوجه روستایی | 0 |  |
| اقتصادی مرغ محلی | | 0 | خسارات ماکیان محلی | 0 | خسارت جوجه محلی | 0 |  |
| خسارات مرغ بومی | | 0 | خسارت ماکیان بومی | 0 | تلفات جوجه بومی | 0 |  |
| خسارات مرغ روستایی | | 0 | خسارت ماکیان روستایی | 0 | تلفات جوجه روستایی | 0 |  |
| خسارات مرغ محلی | | 0 | خسارت ماکیان محلی | 0 | تلفات جوجه محلی | 0 |  |
| خسارت مرغ بومی | | 0 | تلفات ماکیان بومی | 0 | طیور بومی | 0 |  |
| خسارت مرغ روستایی | | 0 | تلفات ماکیان روستایی | 0 | طیور روستایی | 0 |  |
| خسارت مرغ محلی | | 0 | تلفات ماکیان محلی | 0 | طیور محلی | 0 |  |
| تلفات مرغ بومی | | 0 | جوجه بومی | 0 | مدیریت طیور بومی | 0 |  |
| تلفات مرغ روستایی | | 0 | جوجه روستایی | 0 | مدیریت طیور روستایی | 0 |  |
| تلفات مرغ محلی | | 0 | جوجه محلی | 0 | مدیریت طیور محلی | 0 |  |
| ماکیان بومی | | 0 | مدیریت جوجه بومی | 0 | اقتصادی طیور بومی | 0 |  |
| ماکیان روستایی | | 0 | مدیریت جوجه روستایی | 0 | اقتصادی طیور روستایی | 0 |  |
| ماکیان محلی | | 0 | مدیریت جوجه محلی | 0 | اقتصادی طیور محلی | 0 |  |
| خسارات طیور بومی | | 0 | خسارات طیور روستایی | 0 | خسارات طیور محلی | 0 |  |
| خسارت طیور بومی | | 0 | خسارت طیور روستایی | 0 | خسارت طیور محلی | 0 |  |
| تلفات طیور بومی | | 0 | تلفات طیور روستایی | 0 | تلفات طیور محلی | 0 |  |

Table 17-Search and results in the Journal of Animal Production (JAP)/ مجله تولیدات دامی

| Type | Name | Website link | | Search date | | Time-frame | Total hits |
| --- | --- | --- | --- | --- | --- | --- | --- |
| Journal | JAP | <https://jap.ut.ac.ir/> | | 12/12/2021 | | 1998^[[3]](#footnote-3)^-2021 | 5 |
| Search strings | | **Number of hits** | **Search strings** | **Number of hits** | **Search strings** | **Number of hits** |  |
| مرغ بومی | | 5 | اقتصادی ماکیان بومی | 0 | خسارت جوجه بومی | 0 |  |
| مرغ روستایی | | 0 | اقتصادی ماکیان روستایی | 0 | خسارت جوجه روستایی | 0 |  |
| مرغ محلی | | 0 | اقتصادی ماکیان محلی | 0 | خسارت جوجه محلی | 0 |  |
| مدیریت مرغ بومی | | 0 | خسارات ماکیان بومی | 0 | تلفات جوجه بومی | 0 |  |
| مدیریت مرغ روستایی | | 0 | خسارات ماکیان روستایی | 0 | تلفات جوجه روستایی | 0 |  |
| مدیریت مرغ محلی | | 0 | خسارات ماکیان محلی | 0 | تلفات جوجه محلی | 0 |  |
| اقتصادی مرغ بومی | | 0 | خسارت ماکیان بومی | 0 | طیور بومی | 0 |  |
| اقتصادی مرغ روستایی | | 0 | خسارت ماکیان روستایی | 0 | طیور روستایی | 0 |  |
| اقتصادی مرغ محلی | | 0 | خسارت ماکیان روستایی | 0 | طیور محلی | 0 |  |
| خسارات مرغ بومی | | 0 | تلفات ماکیان بومی | 0 | مدیریت طیور بومی | 0 |  |
| خسارات مرغ روستایی | | 0 | تلفات ماکیان روستایی | 0 | مدیریت طیور روستایی | 0 |  |
| خسارات مرغ محلی | | 0 | تلفات ماکیان محلی | 0 | مدیریت طیور محلی | 0 |  |
| خسارت مرغ بومی | | 0 | جوجه بومی | 0 | اقتصادی طیور بومی | 0 |  |
| خسارت مرغ روستایی | | 0 | جوجه روستایی | 0 | اقتصادی طیور روستایی | 0 |  |
| خسارت مرغ محلی | | 0 | جوجه محلی | 0 | اقتصادی طیور محلی | 0 |  |
| تلفات مرغ بومی | | 0 | مدیریت جوجه بومی | 0 | خسارات طیور بومی | 0 |  |
| تلفات مرغ روستایی | | 0 | مدیریت جوجه روستایی | 0 | خسارات طیور روستایی | 0 |  |
| تلفات مرغ محلی | | 0 | مدیریت جوجه محلی | 0 | خسارات طیور محلی | 0 |  |
| ماکیان بومی | | 0 | اقتصادی جوجه بومی | 0 | خسارت طیور بومی | 0 |  |
| ماکیان بومی | | 0 | اقتصادی جوجه روستایی | 0 | خسارت طیور روستایی | 0 |  |
| ماکیان محلی | | 0 | اقتصادی جوجه محلی | 0 | خسارت طیور محلی | 0 |  |
| مدیریت ماکیان بومی | | 0 | خسارات جوجه بومی | 0 | تلفات طیور بومی | 0 |  |
| مدیریت ماکیان روستایی | | 0 | خسارات جوجه روستایی | 0 | تلفات طیور روستایی | 0 |  |
| مدیریت ماکیان محلی | | 0 | خسارات جوجه محلی | 0 | تلفات طیور محلی | 0 |  |

Table 18-Search and results in Iranian Veterinary Journal (IVJ)/ نشریه دامپزشکی ایران

| Type | Name | Website link | | Search date | | Time-frame | Total hits |
| --- | --- | --- | --- | --- | --- | --- | --- |
| Journal | IVJ | <http://www.ivj.ir/> | | 12/12/2021 | | 2003^[[4]](#footnote-4)^-2021 | 6 |
| Search strings | | **Number of hits** | **Search strings** | **Number of hits** | **Search strings** | **Number of hits** |  |
| مرغ بومی | | 4 | مدیریت ماکیان محلی | 0 | خسارات جوجه روستایی | 0 |  |
| مرغ روستایی | | 0 | اقتصادی ماکیان بومی | 0 | خسارات جوجه محلی | 0 |  |
| مرغ محلی | | 0 | اقتصادی ماکیان روستایی | 0 | خسارت جوجه بومی | 0 |  |
| مدیریت مرغ بومی | | 0 | اقتصادی ماکیان محلی | 0 | خسارت جوجه روستایی | 0 |  |
| مدیریت مرغ روستایی | | 0 | خسارات ماکیان بومی | 0 | خسارت جوجه محلی | 0 |  |
| مدیریت مرغ محلی | | 0 | خسارات ماکیان روستایی | 0 | تلفات جوجه بومی | 0 |  |
| اقتصادی مرغ بومی | | 0 | خسارات ماکیان محلی | 0 | تلفات جوجه روستایی | 0 |  |
| اقتصادی مرغ روستایی | | 0 | خسارت ماکیان بومی | 0 | تلفات جوجه محلی | 0 |  |
| اقتصادی مرغ محلی | | 0 | خسارت ماکیان روستایی | 0 | طیور بومی | 0 |  |
| خسارات مرغ بومی | | 0 | خسارت ماکیان محلی | 0 | طیور روستایی | 0 |  |
| خسارات مرغ روستایی | | 0 | تلفات ماکیان بومی | 0 | طیور محلی | 0 |  |
| خسارات مرغ محلی | | 0 | تلفات ماکیان روستایی | 0 | مدیریت طیور بومی | 0 |  |
| خسارت مرغ بومی | | 0 | تلفات ماکیان محلی | 0 | مدیریت طیور روستایی | 0 |  |
| خسارت مرغ روستایی | | 0 | جوجه بومی | 1 | مدیریت طیور محلی | 0 |  |
| خسارت مرغ محلی | | 0 | جوجه روستایی | 0 | اقتصادی طیور بومی | 0 |  |
| تلفات مرغ بومی | | 0 | جوجه محلی | 0 | اقتصادی طیور روستایی | 0 |  |
| تلفات مرغ روستایی | | 0 | مدیریت جوجه بومی | 0 | اقتصادی طیور محلی | 0 |  |
| تلفات مرغ محلی | | 0 | مدیریت جوجه روستایی | 0 | خسارات طیور بومی | 0 |  |
| ماکیان بومی | | 1 | مدیریت جوجه محلی | 0 | خسارات طیور روستایی | 0 |  |
| ماکیان روستایی | | 0 | اقتصادی جوجه بومی | 0 | خسارات طیور محلی | 0 |  |
| ماکیان محلی | | 0 | اقتصادی جوجه روستایی | 0 | خسارت طیور بومی | 0 |  |
| مدیریت ماکیان بومی | | 0 | اقتصادی جوجه محلی | 0 | خسارت طیور روستایی | 0 |  |
| مدیریت ماکیان روستایی | | 0 | خسارات جوجه بومی | 0 | خسارت طیور محلی | 0 |  |
| تلفات طیور بومی | | 0 | تلفات طیور روستایی | 0 | تلفات طیور محلی | 0 |  |

Table 19-Search and results in the Iranian Journal of epidemiology (IRJE)/ مجله تخصصی اپیدمیولوژی ایران

| Type | Name | Website link | | Search date | | Time-frame | Total hits |
| --- | --- | --- | --- | --- | --- | --- | --- |
| Journal | IRJE | <https://irje.tums.ac.ir/> | | 12/12/2021 | | 2005^[[5]](#footnote-5)^-2021 | 3 |
| Search strings | | **Number of hits** | **Search strings** | **Number of hits** | **Search strings** | **Number of hits** |  |
| مرغ بومی | | 0 | مدیریت ماکیان محلی | 0 | خسارات جوجه روستایی | 0 |  |
| مرغ روستایی | | 0 | اقتصادی ماکیان بومی | 0 | خسارات جوجه محلی | 0 |  |
| مرغ محلی | | 0 | اقتصادی ماکیان روستایی | 0 | خسارت جوجه بومی | 0 |  |
| مدیریت مرغ بومی | | 0 | اقتصادی ماکیان محلی | 0 | خسارت جوجه روستایی | 0 |  |
| مدیریت مرغ روستایی | | 0 | خسارات ماکیان بومی | 0 | خسارت جوجه محلی | 0 |  |
| مدیریت مرغ محلی | | 0 | خسارات ماکیان روستایی | 0 | تلفات جوجه بومی | 0 |  |
| اقتصادی مرغ بومی | | 0 | خسارات ماکیان محلی | 0 | تلفات جوجه روستایی | 0 |  |
| اقتصادی مرغ روستایی | | 0 | خسارت ماکیان بومی | 0 | تلفات جوجه محلی | 0 |  |
| اقتصادی مرغ محلی | | 0 | خسارت ماکیان روستایی | 0 | طیور بومی | 2 |  |
| خسارات مرغ بومی | | 0 | خسارت ماکیان محلی | 0 | طیور روستایی | 1 |  |
| خسارات مرغ روستایی | | 0 | تلفات ماکیان بومی | 0 | طیور محلی | 0 |  |
| خسارات مرغ محلی | | 0 | تلفات ماکیان روستایی | 0 | مدیریت طیور بومی | 0 |  |
| خسارت مرغ بومی | | 0 | تلفات ماکیان محلی | 0 | مدیریت طیور روستایی | 0 |  |
| خسارت مرغ روستایی | | 0 | جوجه بومی | 0 | مدیریت طیور محلی | 0 |  |
| خسارت مرغ محلی | | 0 | جوجه روستایی | 0 | اقتصادی طیور بومی | 0 |  |
| تلفات مرغ بومی | | 0 | جوجه محلی | 0 | اقتصادی طیور روستایی | 0 |  |
| تلفات مرغ روستایی | | 0 | مدیریت جوجه بومی | 0 | اقتصادی طیور محلی | 0 |  |
| تلفات مرغ محلی | | 0 | مدیریت جوجه روستایی | 0 | خسارات طیور بومی | 0 |  |
| ماکیان بومی | | 0 | مدیریت جوجه محلی | 0 | خسارات طیور روستایی | 0 |  |
| ماکیان روستایی | | 0 | اقتصادی جوجه بومی | 0 | خسارات طیور محلی | 0 |  |
| ماکیان محلی | | 0 | اقتصادی جوجه روستایی | 0 | خسارت طیور بومی | 0 |  |
| مدیریت ماکیان بومی | | 0 | اقتصادی جوجه محلی | 0 | خسارت طیور روستایی | 0 |  |
| مدیریت ماکیان روستایی | | 0 | خسارات جوجه بومی | 0 | تلفات طیور روستایی | 0 |  |
| خسارت طیور محلی | | 0 | تلفات طیور بومی | 0 | تلفات طیور محلی | 0 |  |

Table 20-Search and results in Iranian Journal of Veterinary Medicine (IJVM) /مجله طب دامی ایران

| Type | Name | Website link | | Search date | | Time-frame | Total hits |
| --- | --- | --- | --- | --- | --- | --- | --- |
| Journal | LJVM | <https://ijvm.ut.ac.ir/> | | 12/12/2021 | | 2007^[[6]](#footnote-6)^-2021 | 1 |
| Search strings | | **Number of hits** | **Search strings** | **Number of hits** | **Search strings** | **Number of hits** |  |
| مرغ بومی | | 0 | اقتصادی ماکیان بومی | 0 | خسارت جوجه بومی | 0 |  |
| مرغ روستایی | | 0 | اقتصادی ماکیان روستایی | 0 | خسارت جوجه روستایی | 0 |  |
| مرغ محلی | | 0 | اقتصادی ماکیان محلی | 0 | خسارت جوجه محلی | 0 |  |
| مدیریت مرغ بومی | | 0 | خسارات ماکیان بومی | 0 | تلفات جوجه بومی | 0 |  |
| مدیریت مرغ روستایی | | 0 | خسارات ماکیان روستایی | 0 | تلفات جوجه روستایی | 0 |  |
| مدیریت مرغ محلی | | 0 | خسارات ماکیان محلی | 0 | تلفات جوجه محلی | 0 |  |
| اقتصادی مرغ بومی | | 0 | خسارت ماکیان بومی | 0 | طیور بومی | 0 |  |
| اقتصادی مرغ روستایی | | 0 | خسارت ماکیان روستایی | 0 | طیور روستایی | 0 |  |
| اقتصادی مرغ محلی | | 0 | خسارت ماکیان محلی | 0 | طیور محلی | 0 |  |
| خسارات مرغ بومی | | 0 | تلفات ماکیان بومی | 0 | مدیریت طیور بومی | 0 |  |
| خسارات مرغ روستایی | | 0 | تلفات ماکیان روستایی | 0 | مدیریت طیور روستایی | 0 |  |
| خسارات مرغ محلی | | 0 | تلفات ماکیان محلی | 0 | مدیریت طیور محلی | 0 |  |
| خسارت مرغ بومی | | 0 | جوجه بومی | 0 | اقتصادی طیور بومی | 0 |  |
| خسارت مرغ روستایی | | 0 | جوجه روستایی | 0 | اقتصادی طیور روستایی | 0 |  |
| خسارت مرغ محلی | | 0 | جوجه محلی | 0 | اقتصادی طیور محلی | 0 |  |
| تلفات مرغ بومی | | 0 | مدیریت جوجه بومی | 0 | خسارات طیور بومی | 0 |  |
| تلفات مرغ روستایی | | 0 | مدیریت جوجه روستایی | 0 | خسارات طیور روستایی | 0 |  |
| تلفات مرغ محلی | | 0 | مدیریت جوجه محلی | 0 | خسارات طیور محلی | 0 |  |
| ماکیان بومی | | 1 | اقتصادی جوجه بومی | 0 | خسارت طیور بومی | 0 |  |
| ماکیان روستایی | | 0 | اقتصادی جوجه روستایی | 0 | خسارت طیور روستایی | 0 |  |
| ماکیان محلی | | 0 | اقتصادی جوجه محلی | 0 | خسارت طیور محلی | 0 |  |
| مدیریت ماکیان بومی | | 0 | خسارات جوجه بومی | 0 | تلفات طیور بومی | 0 |  |
| مدیریت ماکیان روستایی | | 0 | خسارات جوجه روستایی | 0 | تلفات طیور روستایی | 0 |  |
| مدیریت ماکیان محلی | | 0 | خسارات جوجه محلی | 0 | تلفات طیور محلی | 0 |  |

Table 21-Search and results in the Iranian Journal of Animal Science Research (IJASR)/ نشریه علمی پژوهش‌های علوم دامی ایران

| Type | Name | Website link | | Search date | | Time-frame | Total hits |
| --- | --- | --- | --- | --- | --- | --- | --- |
| Journal | IJASR | <https://ijasr.um.ac.ir/> | | 12/12/2021 | | 2009^[[7]](#footnote-7)^-2021 | 5 |
| Search strings | | **Number of hits** | **Search strings** | **Number of hits** | **Search strings** | **Number of hits** |  |
| مرغ بومی | | 4 | اقتصادی ماکیان بومی | 0 | خسارت جوجه بومی | 0 |  |
| مرغ روستایی | | 0 | اقتصادی ماکیان روستایی | 0 | خسارت جوجه روستایی | 0 |  |
| مرغ محلی | | 0 | اقتصادی ماکیان محلی | 0 | خسارت جوجه محلی | 0 |  |
| مدیریت مرغ بومی | | 0 | خسارات ماکیان بومی | 0 | تلفات جوجه بومی | 0 |  |
| مدیریت مرغ روستایی | | 0 | خسارات ماکیان روستایی | 0 | تلفات جوجه روستایی | 0 |  |
| مدیریت مرغ محلی | | 0 | خسارات ماکیان محلی | 0 | تلفات جوجه محلی | 0 |  |
| اقتصادی مرغ بومی | | 0 | خسارت ماکیان بومی | 0 | طیور بومی | 0 |  |
| اقتصادی مرغ روستایی | | 0 | خسارت ماکیان روستایی | 0 | طیور روستایی | 0 |  |
| اقتصادی مرغ محلی | | 0 | خسارت ماکیان محلی | 0 | طیور محلی | 0 |  |
| خسارات مرغ بومی | | 0 | تلفات ماکیان بومی | 0 | مدیریت طیور بومی | 0 |  |
| خسارات مرغ روستایی | | 0 | تلفات ماکیان روستایی | 0 | مدیریت طیور روستایی | 0 |  |
| خسارات مرغ محلی | | 0 | تلفات ماکیان محلی | 0 | مدیریت طیور محلی | 0 |  |
| خسارت مرغ بومی | | 0 | جوجه بومی | 1 | اقتصادی طیور بومی | 0 |  |
| خسارت مرغ روستایی | | 0 | جوجه روستایی | 0 | اقتصادی طیور روستایی | 0 |  |
| خسارت مرغ محلی | | 0 | جوجه محلی | 0 | اقتصادی طیور محلی | 0 |  |
| تلفات مرغ بومی | | 0 | مدیریت جوجه بومی | 0 | خسارات طیور بومی | 0 |  |
| تلفات مرغ روستایی | | 0 | مدیریت جوجه روستایی | 0 | خسارات طیور روستایی | 0 |  |
| تلفات مرغ محلی | | 0 | مدیریت جوجه محلی | 0 | خسارات طیور محلی | 0 |  |
| ماکیان بومی | | 0 | اقتصادی جوجه بومی | 0 | خسارت طیور بومی | 0 |  |
| ماکیان روستایی | | 0 | اقتصادی جوجه روستایی | 0 | خسارت طیور روستایی | 0 |  |
| ماکیان محلی | | 0 | اقتصادی جوجه محلی | 0 | خسارت طیور محلی | 0 |  |
| مدیریت ماکیان بومی | | 0 | خسارات جوجه بومی | 0 | تلفات طیور بومی | 0 |  |
| مدیریت ماکیان روستایی | | 0 | خسارات جوجه روستایی | 0 | تلفات طیور روستایی | 0 |  |
| مدیریت ماکیان محلی | | 0 | خسارات جوجه محلی | 0 | تلفات طیور محلی | 0 |  |

Table 22- Search and results in the Animal Science Journal (ASJ)/نشریه علوم دامی جهاد کشاورزی

| Type | Name | Website link | | Search date | | Time-frame | Total hits |
| --- | --- | --- | --- | --- | --- | --- | --- |
| Journal | ASJ | <https://asj.areeo.ac.ir/> | | 12/12/2021 | | 1987^[[8]](#footnote-8)^-2021 | 6 |
| Search strings | | **Number of hits** | **Search strings** | **Number of hits** | **Search strings** | **Number of hits** |  |
| مرغ بومی | | 6 | اقتصادی ماکیان بومی | 0 | خسارت جوجه بومی | 0 |  |
| مرغ روستایی | | 0 | اقتصادی ماکیان روستایی | 0 | خسارت جوجه روستایی | 0 |  |
| مرغ محلی | | 0 | اقتصادی ماکیان محلی | 0 | خسارت جوجه محلی | 0 |  |
| مدیریت مرغ بومی | | 0 | خسارات ماکیان بومی | 0 | تلفات جوجه بومی | 0 |  |
| مدیریت مرغ روستایی | | 0 | خسارات ماکیان روستایی | 0 | تلفات جوجه روستایی | 0 |  |
| مدیریت مرغ محلی | | 0 | خسارات ماکیان محلی | 0 | تلفات جوجه محلی | 0 |  |
| اقتصادی مرغ بومی | | 0 | خسارت ماکیان بومی | 0 | طیور بومی | 0 |  |
| اقتصادی مرغ روستایی | | 0 | خسارت ماکیان روستایی | 0 | طیور روستایی | 0 |  |
| اقتصادی مرغ محلی | | 0 | خسارت ماکیان محلی | 0 | طیور محلی | 0 |  |
| خسارات مرغ بومی | | 0 | تلفات ماکیان بومی | 0 | مدیریت طیور بومی | 0 |  |
| خسارات مرغ روستایی | | 0 | تلفات ماکیان روستایی | 0 | مدیریت طیور روستایی | 0 |  |
| خسارات مرغ محلی | | 0 | تلفات ماکیان محلی | 0 | مدیریت طیور محلی | 0 |  |
| خسارت مرغ بومی | | 0 | جوجه بومی | 0 | اقتصادی طیور بومی | 0 |  |
| خسارت مرغ روستایی | | 0 | جوجه روستایی | 0 | اقتصادی طیور روستایی | 0 |  |
| خسارت مرغ محلی | | 0 | جوجه محلی | 0 | اقتصادی طیور محلی | 0 |  |
| تلفات مرغ بومی | | 0 | مدیریت جوجه بومی | 0 | خسارات طیور بومی | 0 |  |
| تلفات مرغ روستایی | | 0 | مدیریت جوجه روستایی | 0 | خسارات طیور روستایی | 0 |  |
| تلفات مرغ محلی | | 0 | مدیریت جوجه محلی | 0 | خسارات طیور محلی | 0 |  |
| ماکیان بومی | | 0 | اقتصادی جوجه بومی | 0 | خسارت طیور بومی | 0 |  |
| ماکیان روستایی | | 0 | اقتصادی جوجه روستایی | 0 | خسارت طیور روستایی | 0 |  |
| ماکیان محلی | | 0 | اقتصادی جوجه محلی | 0 | خسارت طیور محلی | 0 |  |
| مدیریت ماکیان بومی | | 0 | خسارات جوجه بومی | 0 | تلفات طیور بومی | 0 |  |
| مدیریت ماکیان روستایی | | 0 | خسارات جوجه روستایی | 0 | تلفات طیور روستایی | 0 |  |
| مدیریت ماکیان محلی | | 0 | خسارات جوجه محلی | 0 | تلفات طیور محلی | 0 |  |

Table 23- Search and results in the Applied Animal Science Research Journal (ASRIP)/فصلنامه تحقیقات کاربردی درعلوم دامی

| Type | Name | Website link | | Search date | | Time-frame | Total hits |
| --- | --- | --- | --- | --- | --- | --- | --- |
| Journal | ASRIP | <https://asrip.areeo.ac.ir/> | | 12/12/2021 | | 2011^[[9]](#footnote-9)^-2021 | 0 |
| Search strings | | **Number of hits** | **Search strings** | **Number of hits** | **Search strings** | **Number of hits** |  |
| مرغ بومی | | 0 | مدیریت ماکیان محلی | 0 | خسارات جوجه محلی | 0 |  |
| مرغ روستایی | | 0 | اقتصادی ماکیان بومی | 0 | خسارت جوجه بومی | 0 |  |
| مرغ محلی | | 0 | اقتصادی ماکیان روستایی | 0 | خسارت جوجه روستایی | 0 |  |
| مدیریت مرغ بومی | | 0 | اقتصادی ماکیان محلی | 0 | خسارت جوجه محلی | 0 |  |
| مدیریت مرغ روستایی | | 0 | خسارات ماکیان بومی | 0 | تلفات جوجه بومی | 0 |  |
| مدیریت مرغ محلی | | 0 | خسارات ماکیان روستایی | 0 | تلفات جوجه روستایی | 0 |  |
| اقتصادی مرغ بومی | | 0 | خسارات ماکیان محلی | 0 | تلفات جوجه محلی | 0 |  |
| اقتصادی مرغ روستایی | | 0 | خسارت ماکیان بومی | 0 | طیور بومی | 0 |  |
| اقتصادی مرغ محلی | | 0 | خسارت ماکیان روستایی | 0 | طیور روستایی | 0 |  |
| خسارات مرغ بومی | | 0 | خسارت ماکیان محلی | 0 | طیور محلی | 0 |  |
| خسارات مرغ روستایی | | 0 | تلفات ماکیان بومی | 0 | مدیریت طیور بومی | 0 |  |
| خسارات مرغ محلی | | 0 | تلفات ماکیان روستایی | 0 | مدیریت طیور روستایی | 0 |  |
| خسارت مرغ بومی | | 0 | تلفات ماکیان محلی | 0 | مدیریت طیور محلی | 0 |  |
| خسارت مرغ روستایی | | 0 | جوجه بومی | 0 | اقتصادی طیور بومی | 0 |  |
| خسارت مرغ محلی | | 0 | جوجه روستایی | 0 | اقتصادی طیور روستایی | 0 |  |
| تلفات مرغ بومی | | 0 | جوجه محلی | 0 | اقتصادی طیور محلی | 0 |  |
| تلفات مرغ روستایی | | 0 | مدیریت جوجه بومی | 0 | خسارات طیور بومی | 0 |  |
| تلفات مرغ محلی | | 0 | مدیریت جوجه روستایی | 0 | خسارات طیور روستایی | 0 |  |
| ماکیان بومی | | 0 | مدیریت جوجه محلی | 0 | خسارات طیور محلی | 0 |  |
| ماکیان روستایی | | 0 | اقتصادی جوجه بومی | 0 | خسارت طیور بومی | 0 |  |
| ماکیان محلی | | 0 | اقتصادی جوجه روستایی | 0 | خسارت طیور روستایی | 0 |  |
| مدیریت ماکیان بومی | | 0 | خسارات جوجه بومی | 0 | خسارت طیور محلی | 0 |  |
| مدیریت ماکیان روستایی | | 0 | خسارات جوجه روستایی | 0 | تلفات طیور بومی | 0 |  |
| تلفات طیور روستایی | | 0 | تلفات طیور محلی | 0 |  |  |  |

Table 24- Search and results in the Animal Production Research (AR)/ فصلنامه تحقیقات علوم دامی دانشگاه گیلان

| Type | Name | Website link | | Search date | | Time-frame | Total hits |
| --- | --- | --- | --- | --- | --- | --- | --- |
| Journal | AR | <https://ar.guilan.ac.ir/> | | 12/12/2021 | | 2012^[[10]](#footnote-10)^-2021 | 3 |
| Search strings | | **Number of hits** | **Search strings** | **Number of hits** | **Search strings** | **Number of hits** |  |
| مرغ بومی | | 3 | مدیریت ماکیان محلی | 0 | خسارات جوجه روستایی | 0 |  |
| مرغ روستایی | | 0 | مدیریت ماکیان محلی | 0 | خسارات جوجه محلی | 0 |  |
| مرغ محلی | | 0 | اقتصادی ماکیان روستایی | 0 | خسارت جوجه بومی | 0 |  |
| مدیریت مرغ بومی | | 0 | اقتصادی ماکیان محلی | 0 | خسارت جوجه روستایی | 0 |  |
| مدیریت مرغ روستایی | | 0 | خسارات ماکیان بومی | 0 | خسارت جوجه محلی | 0 |  |
| مدیریت مرغ محلی | | 0 | خسارات ماکیان روستایی | 0 | تلفات جوجه بومی | 0 |  |
| اقتصادی مرغ بومی | | 0 | خسارات ماکیان محلی | 0 | تلفات جوجه روستایی | 0 |  |
| اقتصادی مرغ روستایی | | 0 | خسارت ماکیان بومی | 0 | تلفات جوجه محلی | 0 |  |
| اقتصادی مرغ محلی | | 0 | خسارت ماکیان روستایی | 0 | طیور بومی | 0 |  |
| خسارات مرغ بومی | | 0 | خسارت ماکیان محلی | 0 | طیور روستایی | 0 |  |
| خسارات مرغ روستایی | | 0 | تلفات ماکیان بومی | 0 | طیور محلی | 0 |  |
| خسارات مرغ محلی | | 0 | تلفات ماکیان روستایی | 0 | مدیریت طیور بومی | 0 |  |
| خسارت مرغ بومی | | 0 | تلفات ماکیان محلی | 0 | مدیریت طیور روستایی | 0 |  |
| خسارت مرغ روستایی | | 0 | جوجه بومی | 0 | مدیریت طیور محلی | 0 |  |
| خسارت مرغ محلی | | 0 | جوجه روستایی | 0 | اقتصادی طیور بومی | 0 |  |
| تلفات مرغ بومی | | 0 | جوجه محلی | 0 | اقتصادی طیور روستایی | 0 |  |
| تلفات مرغ روستایی | | 0 | مدیریت جوجه بومی | 0 | اقتصادی طیور محلی | 0 |  |
| تلفات مرغ محلی | | 0 | مدیریت جوجه روستایی | 0 | خسارات طیور بومی | 0 |  |
| ماکیان بومی | | 0 | مدیریت جوجه محلی | 0 | خسارات طیور روستایی | 0 |  |
| ماکیان روستایی | | 0 | اقتصادی جوجه بومی | 0 | خسارات طیور محلی | 0 |  |
| ماکیان محلی | | 0 | اقتصادی جوجه روستایی | 0 | خسارت طیور بومی | 0 |  |
| مدیریت ماکیان بومی | | 0 | اقتصادی جوجه محلی | 0 | خسارت طیور روستایی | 0 |  |
| مدیریت ماکیان روستایی | | 0 | خسارات جوجه بومی | 0 | خسارت طیور محلی | 0 |  |
| تلفات طیور بومی | | 0 | تلفات طیور روستایی | 0 | تلفات طیور محلی | 0 |  |

Table 25-Search and results in the Journal of Animal Science Research/ نشریه پژوهش‌های علوم دامی دانشگاه تبریز

| Type | Name | | Website link | | Search date | | Time-frame | Total hits |
| --- | --- | --- | --- | --- | --- | --- | --- | --- |
| Journal | Animal Science | | <https://animalscience.tabrizu.ac.ir/> | | 12/12/2021 | | 1990^[[11]](#footnote-11)^-2021 | 3 |
| Search strings | | **Number of hits** | | **Search strings** | **Number of hits** | **Search strings** | **Number of hits** |  |
| مرغ بومی | | 2 | | ماکیان محلی | 0 | مدیریت جوجه روستایی | 0 |  |
| مرغ روستایی | | 0 | | مدیریت ماکیان بومی | 0 | مدیریت جوجه محلی | 0 |  |
| مرغ محلی | | 0 | | مدیریت ماکیان روستایی | 0 | اقتصادی جوجه بومی | 0 |  |
| مدیریت مرغ بومی | | 0 | | مدیریت ماکیان محلی | 0 | اقتصادی جوجه روستایی | 0 |  |
| مدیریت مرغ روستایی | | 0 | | اقتصادی ماکیان بومی | 0 | اقتصادی جوجه محلی | 0 |  |
| مدیریت مرغ محلی | | 0 | | اقتصادی ماکیان روستایی | 0 | خسارات جوجه بومی | 0 |  |
| اقتصادی مرغ بومی | | 0 | | اقتصادی ماکیان محلی | 0 | خسارات جوجه روستایی | 0 |  |
| اقتصادی مرغ روستایی | | 0 | | خسارات ماکیان بومی | 0 | خسارات جوجه محلی | 0 |  |
| اقتصادی مرغ محلی | | 0 | | خسارات ماکیان روستایی | 0 | خسارت جوجه بومی | 0 |  |
| خسارات مرغ بومی | | 0 | | خسارات ماکیان محلی | 0 | خسارت جوجه روستایی | 0 |  |
| خسارات مرغ روستایی | | 0 | | خسارت ماکیان بومی | 0 | خسارت جوجه محلی | 0 |  |
| خسارات مرغ محلی | | 0 | | خسارت ماکیان روستایی | 0 | تلفات جوجه بومی | 0 |  |
| خسارت مرغ بومی | | 0 | | خسارت ماکیان محلی | 0 | تلفات جوجه روستایی | 0 |  |
| خسارت مرغ روستایی | | 0 | | تلفات ماکیان بومی | 0 | تلفات جوجه محلی | 0 |  |
| خسارت مرغ محلی | | 0 | | تلفات ماکیان روستایی | 0 | طیور بومی | 1 |  |
| تلفات مرغ بومی | | 0 | | تلفات ماکیان محلی | 0 | طیور روستایی | 0 |  |
| تلفات مرغ روستایی | | 0 | | جوجه بومی | 0 | طیور محلی | 0 |  |
| تلفات مرغ محلی | | 0 | | جوجه روستایی | 0 | مدیریت طیور بومی | 0 |  |
| ماکیان بومی | | 0 | | جوجه محلی | 0 | مدیریت طیور روستایی | 0 |  |
| ماکیان روستایی | | 0 | | مدیریت جوجه بومی | 0 | مدیریت طیور محلی | 0 |  |
| اقتصادی طیور روستایی | | 0 | | اقتصادی طیور محلی | 0 | اقتصادی طیور بومی | 0 |  |
| خسارات طیور بومی | | 0 | | خسارات طیور روستایی | 0 | خسارات طیور محلی | 0 |  |
| خسارت طیور بومی | | 0 | | خسارت طیور روستایی | 0 | خسارت طیور محلی | 0 |  |
| تلفات طیور بومی | | 0 | | تلفات طیور روستایی | 0 | تلفات طیور محلی | 0 |  |

1. The Journal was launched in 1988 [↑](#footnote-ref-1)
2. Journal was launched in 2012 [↑](#footnote-ref-2)
3. Journal was launched in 1998 [↑](#footnote-ref-3)
4. Journal was launched in 2003 [↑](#footnote-ref-4)
5. Journal was launched in 2005 [↑](#footnote-ref-5)
6. Journal was launched in 2007 [↑](#footnote-ref-6)
7. Journal was launched in 2009 [↑](#footnote-ref-7)
8. Journal was launched in 1987 [↑](#footnote-ref-8)
9. Journal was launched in 2011 [↑](#footnote-ref-9)
10. Journal was launched in 2012 [↑](#footnote-ref-10)
11. Journal was launched in 1990 [↑](#footnote-ref-11)
