# Supplementary material for: Economic impact of chicken diseases and other causes of morbidity or mortality in backyard farms in low-income and middle-income countries: a systematic review and meta-analysis
Source: BMC Vet Res. 2025 Mar 7;21:151. doi: 10.1186/s12917-025-04549-7 (PMC11887245; doi:10.1186/s12917-025-04549-7)
Supplement: Supplementary file 2 — Additional file 2. Details of the economic analysis. [file 12917_2025_4549_MOESM2_ESM.docx]

# Details of the economic analysis

## Price conversion in each country

- No CPI data were available for 2021 onwards in Uganda and Tanzania and therefore, for these two countries, prices that were captured in subsequent years than 2020 (i.e., 2021 and 2022), were considered to be from 2020 as CPI data of the base year were not available.
- The African Financial Community (CFA) Franc is the currency for several countries in the study: Burkina Faso, Cameroon, Ivory Coast and Senegal.

## Estimation of broiler and layer prices in each country

- Countries for which the DOC price of a broiler chicken for that country was used as the DOC price of a layer hen: Bangladesh, Burkina Faso, Cambodia, Cameroon, Ecuador, Ethiopia, Guinea, Haiti, India, Iraq, Madagascar, Nicaragua, Senegal, South Africa, Sri Lanka, Sudan, Tanzania, Thailand and Vietnam.
- Countries for which prices were not found and therefore they (prices) were borrowed from a neighbouring country with a similar purchasing power gross domestic product per head: Burkina Faso (DOC price of a broiler borrowed from Ghana), Haiti (DOC price of a broiler and a broiler chicken price borrowed from the Dominican Republic), Haiti (chicken price borrowed from the Dominican Republic), Madagascar (DOC price of a broiler borrowed from Zimbabwe) and Pakistan (price of a layer hen borrowed from India).
