# Supplementary material for: Economic impact of chicken diseases and other causes of morbidity or mortality in backyard farms in low-income and middle-income countries: a systematic review and meta-analysis
Source: BMC Vet Res. 2025 Mar 7;21:151. doi: 10.1186/s12917-025-04549-7 (PMC11887245; doi:10.1186/s12917-025-04549-7)

# Forest plots of meta-analyses by mortality cause

Note that the same study might be cited more than once in the meta-analyses. The reason for this is because one study can report several mortality causes.

## Non-infectious causes

### Predation


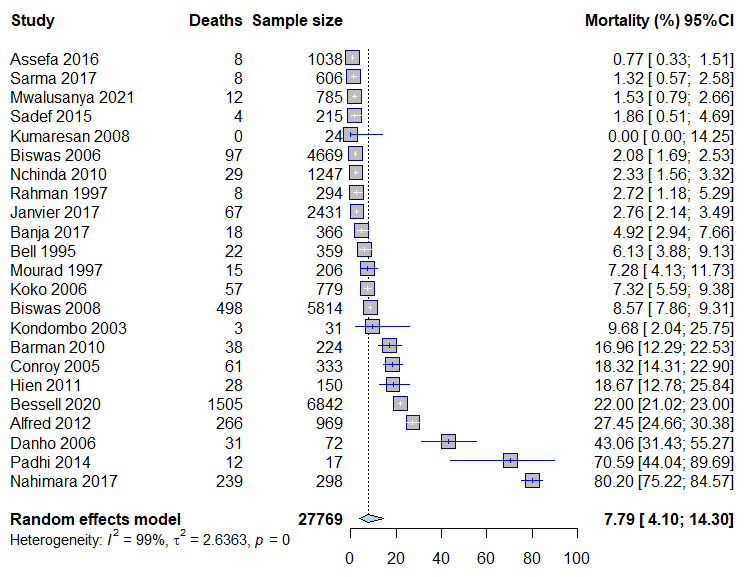


### Cachexia


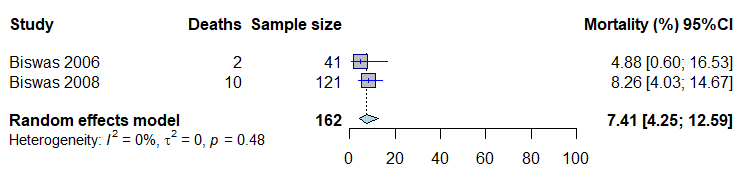


### Weather conditions


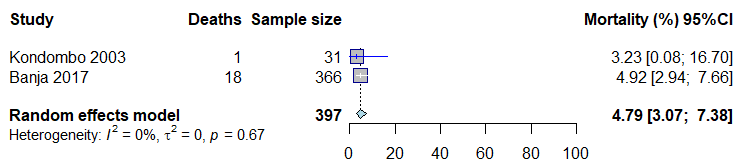


### Injuries


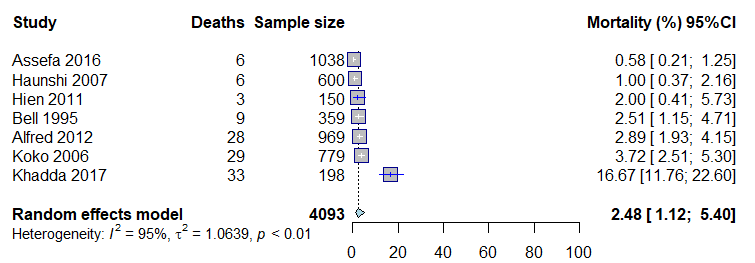


## Infectious causes

### Bacterial diseases


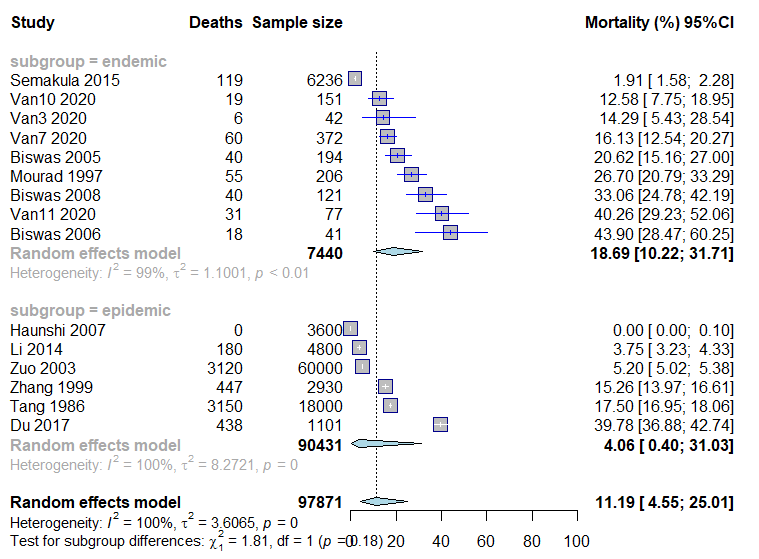


### Bacterial and parasitic diseases


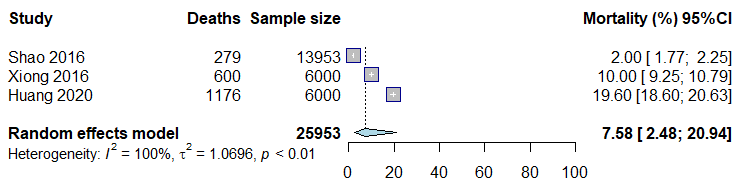


### Bacterial and viral diseases


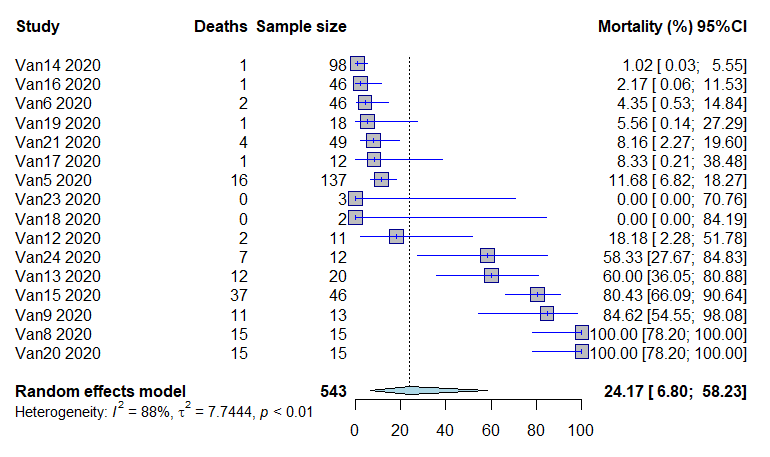


### Viral diseases


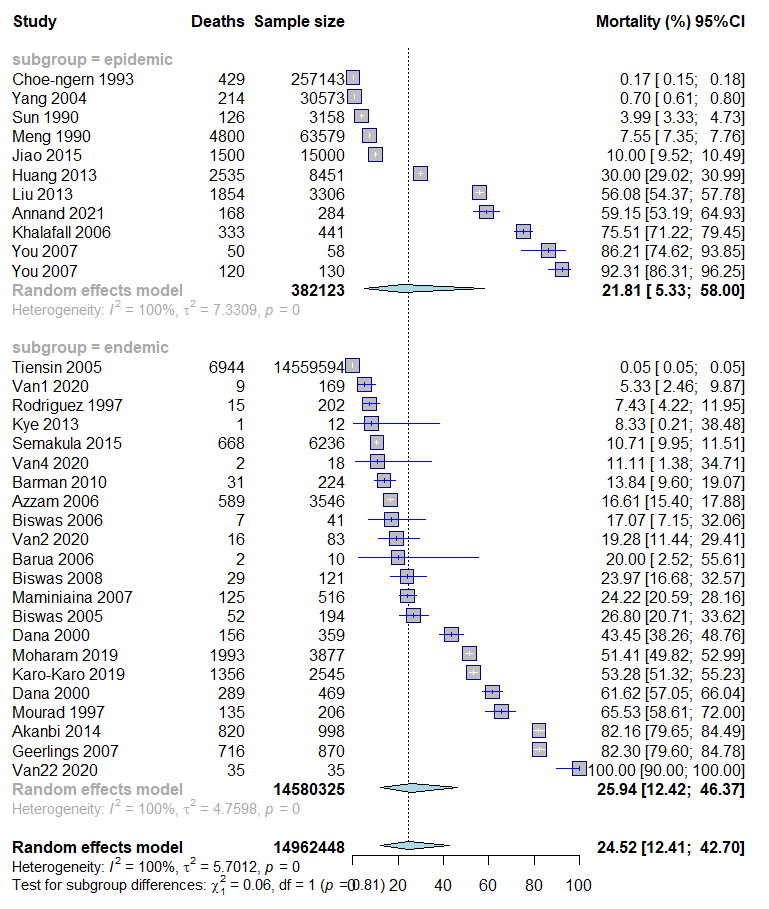


### Parasitic diseases


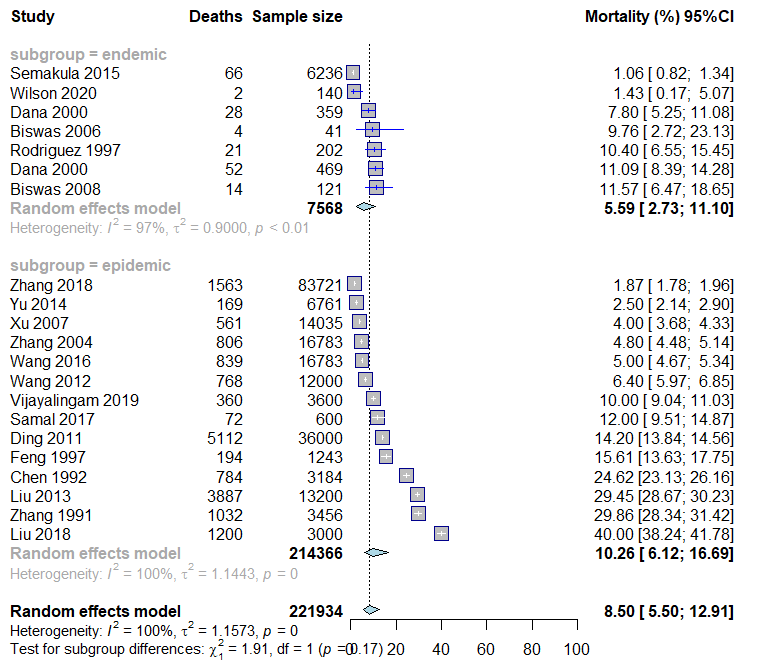


### Fungal diseases


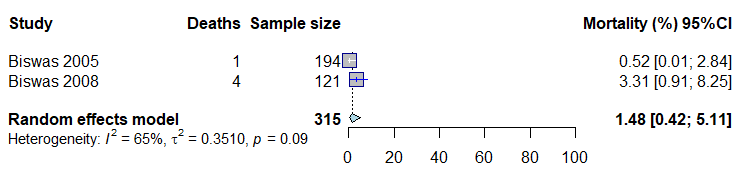

Supplement: Supplementary file 3 — Additional file 3. Forest plots of meta-analyses by mortality cause. [file 12917_2025_4549_MOESM3_ESM.docx]
