# Supplementary material for: Economic impact of chicken diseases and other causes of morbidity or mortality in backyard farms in low-income and middle-income countries: a systematic review and meta-analysis
Source: BMC Vet Res. 2025 Mar 7;21:151. doi: 10.1186/s12917-025-04549-7 (PMC11887245; doi:10.1186/s12917-025-04549-7)

# Forest-plots of meta-analysis of economic losses by mortality cause

## Infectious causes

### Bacteria


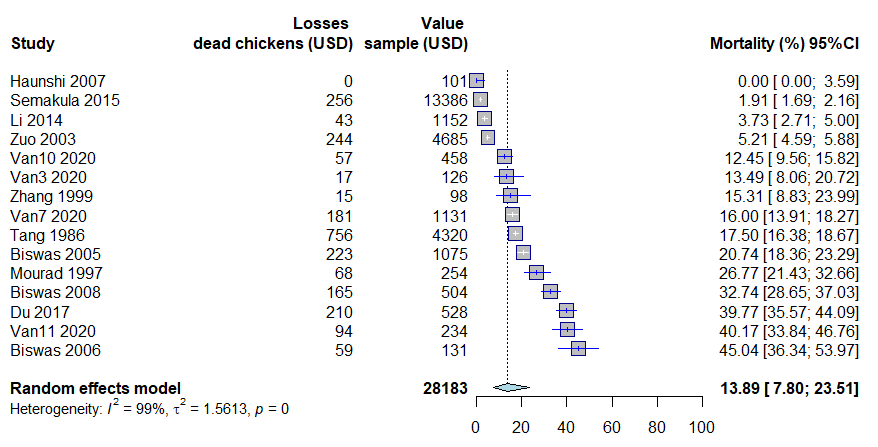


### Bacteria and viral diseases


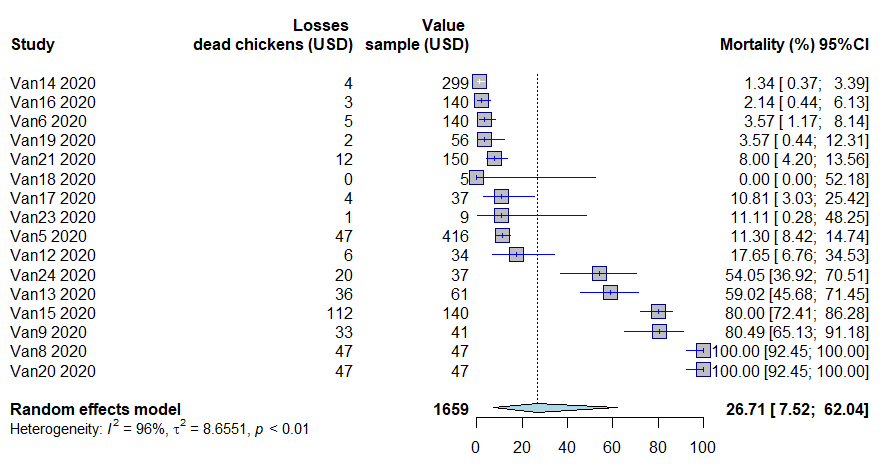


### Bacteria and parasite diseases


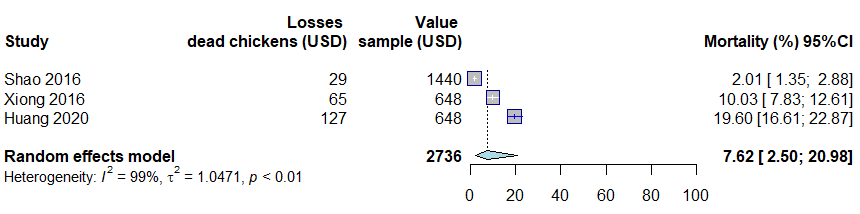


### Parasite diseases


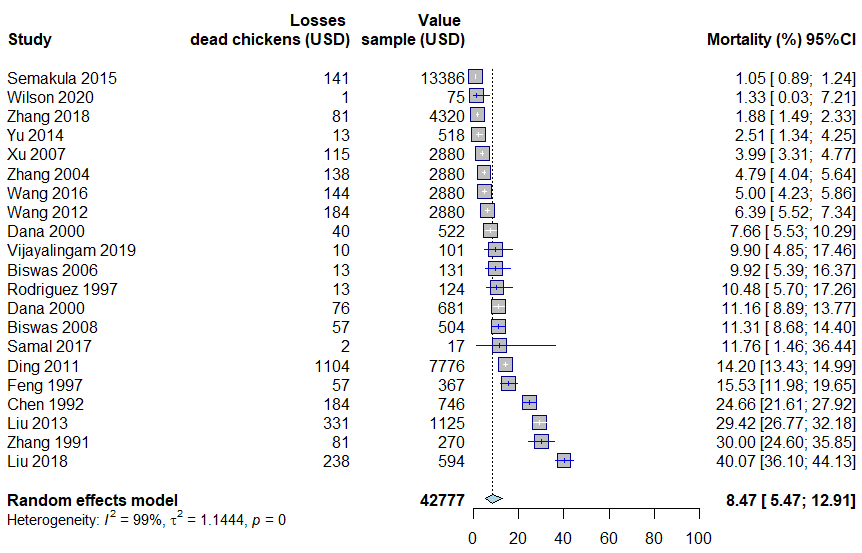


### Viral diseases


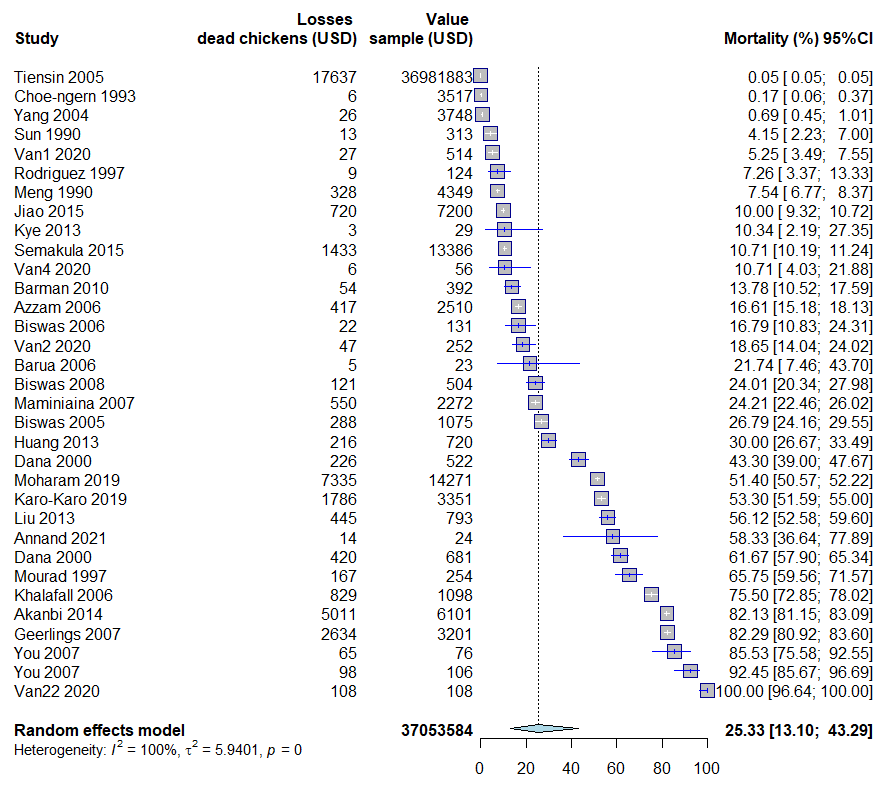


### Fungus


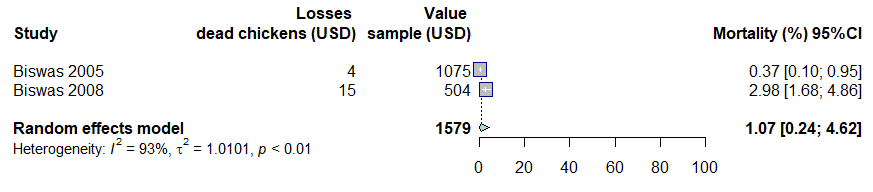


## Non-infectious causes

### Predation


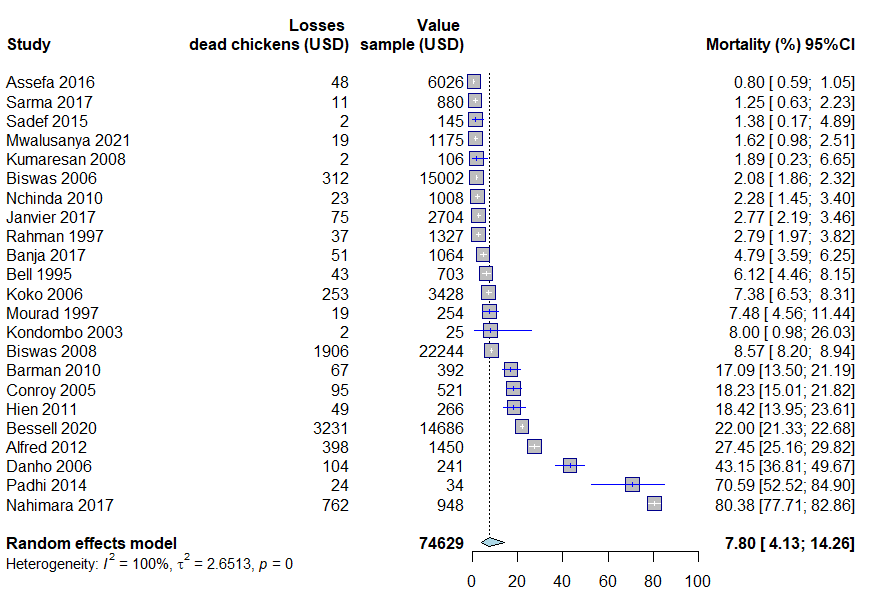


### Injuries


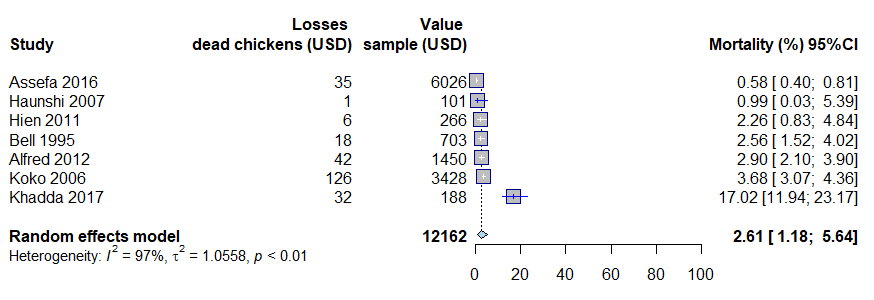


### Cachexia


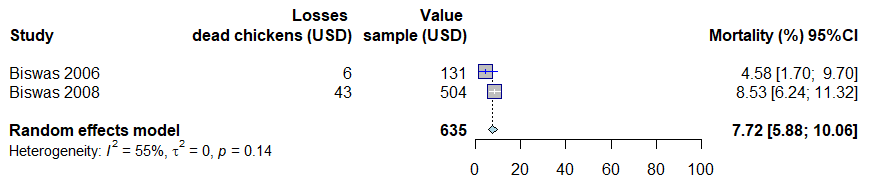


### Weather


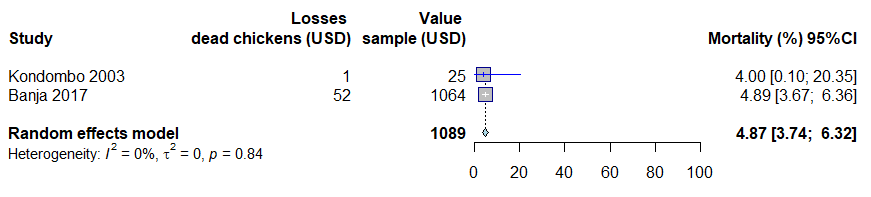

Supplement: Supplementary file 5 — Additional file 5. Forest plots of the meta-analysis of economic losses by mortality cause. [file 12917_2025_4549_MOESM5_ESM.docx]
