# Supplementary material for: Economic impact of chicken diseases and other causes of morbidity or mortality in backyard farms in low-income and middle-income countries: a systematic review and meta-analysis
Source: BMC Vet Res. 2025 Mar 7;21:151. doi: 10.1186/s12917-025-04549-7 (PMC11887245; doi:10.1186/s12917-025-04549-7)
Supplement: Supplementary file 6 — Additional file 6. References to selected studies for the systematic literature review. [file 12917_2025_4549_MOESM6_ESM.docx]

# References of selected studies for the systematic literature review

Akanbi, O. B., & Taiwo, V. O. (2014). Backyard Poultry Mortality Associated with Highly Pathogenic Avian Influenza (HPAI) H5N1 outbreaks in Nigeria. *IOSR Journal of Agriculture and Veterinary Science*, *7*(9), 23–27. https://doi.org/10.9790/2380-07922327

Alfred, B., Msoffe, P. L. M., Kajuna, F. F., Bunn, D., Muhairwa, A. P., & Cardona, C. J. (2012). Causes of losses in free range local chickens following control of Newcastle disease in three villages in Morogoro, Tanzania. *Livestock Research for Rural Development*, *24*(7). http://lrrd.cipav.org.co/lrrd24/7/alfr24124.htm

Annand, E. J., High, H., Wong, F. Y. K., Phommachanh, P., Chanthavisouk, C., Happold, J., Dhingra, M. S., Eagles, D., Britton, P. N., & Alders, R. G. (2021). Detection of highly pathogenic avian influenza in Sekong Province Lao PDR 2018—Potential for improved surveillance and management in endemic regions. *Transboundary and Emerging Diseases*, *68*(1), 168–182. https://doi.org/10.1111/tbed.13673

Assefa, H., Bogale, A., Gebremedhin, B., Mekuriaw, Z., Derso, T., Dessalegn, Y., Tegegne, A., & Hoekstra, D. (2016). Village Chicken Production and Marketing in West Gojjam Zone, Ethiopia. *Current Research in Agricultural Sciences*, *3*(4), 64–73. https://doi.org/10.18488/journal.68/2016.3.4/68.4.64.73

Azzam, A. H., Youseif, H. M. Z., & Ahmed, E. E. K. (2006). Prevalence of Gumboro disease in vaccinated and non-vaccinated village chickens in Egypt. In IAEA (Ed.), *Improving farmyard poultry production in Africa: Interventions and their economic assessment* (p. 280). IAEA. https://www-pub.iaea.org/mtcd/publications/pdf/te_1489_web.pdf

Banja, B. K., Ananth, P. N., Singh, S., Behera, S., & Jayasankar, P. (2017). A study on the Frontline demonstration of backyard poultry in rural Odisha. *Livestock Research for Rural Development*, *29*(5). https://lrrd.cipav.org.co/lrrd29/5/anan29087.html

Barman, L., Flensburg, M., Permin, A., Madsen, M., & Islam, M. (2010). A controlled study to assess the effects of vaccination against Newcastle disease in village chickens. *Bangladesh Veterinarian*, *27*(2), 56–61. https://doi.org/10.3329/bvet.v27i2.7555

Barua, H., Biswas, P. K., Anwar, M. N., Dey, B. C., & Debnath, M. C. (2006). Serosurvey and Isolation of Infectious Bronchitis Virus in Chickens Reared in Commercial and Semi-Scavenging Systems. *Bangladesh Journal of Microbiology*, *23*(2), 114–117. https://doi.org/10.3329/bjm.v23i2.873

Bell, J. G., Fotzo, T. M., Amara, A., & Agbede, G. (1995). A field trial of the heat resistant V4 vaccine against Newcastle disease by eye-drop inoculation in village poultry in Cameroon. *Preventive Veterinary Medicine*, *25*(1), 19–25. https://doi.org/10.1016/0167-5877(95)00494-7

Bessell, P. R., Woolley, R., Stevenson, S., Al-Riyami, L., Opondo, P., Lai, L., & Gammon, N. (2020). An analysis of the impact of Newcastle disease vaccination and husbandry practice on smallholder chicken productivity in Uganda. *Preventive Veterinary Medicine*, *177*(March), 104975. https://doi.org/10.1016/j.prevetmed.2020.104975

Bhuiyan, A. R., Lauridsen, C., Howlider, A. R., & Jakobsen, K. (2004). Importance of vitamin A supplementation for performance of Sonali chickens under smallholder farm conditions in a tropical climate. *Livestock Research for Rural Development*, *16*(10).

Biswas, P. K., Biswas, D., Ahmed, S., Rahman, A., & Debnath, N. C. (2005). A longitudinal study of the incidence of major endemic and epidemic diseases affecting semi-scavenging chickens reared under the Participatory Livestock Development Project areas in Bangladesh. *Avian Pathology*, *34*(4), 303–312. https://doi.org/10.1080/03079450500178972

Biswas, P. K., Uddin, G. M. N., Barua, H., Roy, K., Biswas, D., Ahad, A., & Debnath, N. C. (2006). Causes of loss of Sonali chickens on smallholder households in Bangladesh. *Preventive Veterinary Medicine*, *76*(3–4), 185–195. https://doi.org/10.1016/j.prevetmed.2006.05.001

Biswas, P. K., Uddin, G. M. N., Barua, H., Roy, K., Biswas, D., Ahad, A., & Debnath, N. C. (2008). Survivability and causes of loss of broody-hen chicks on smallholder households in Bangladesh. *Preventive Veterinary Medicine*, *83*(3–4), 260–271. https://doi.org/10.1016/j.prevetmed.2007.08.001

Chen. (1992). Diagnosis and treatment of chicken ascariasis (EN). *Gansu Animal Husbandry and Veterinary*. https://chn.oversea.cnki.net/KCMS/detail/detail.aspx?dbcode=CJFD&dbname=CJFD9093&filename=GXMS199206035&uniplatform=OVERSEA&v=WWEZtWxQXOCXXPEckL9wZRG5hnU3ZJOtWJji57VhLEoOli97iB54-VdJfBXp8Rys

Choe-ngern, N., Antrasen, C., Suksaithaichana, P., & Aocharoen, B. (1993). Infectious laryngitis in laying hens in Southern Thailand (EN). *Songklanakarin Journal of Science and Technology*.

Conroy, C., Sparks, N., Chandrasekaran, D., Sharma, A., Shindey, D., Singh, L. R., Natarajan, A., & Anitha, K. (2005). The significance of predation as a constraint in scavenging poultry systems: some findings from India. *Livestock Research for Rural Development*, *17*(6). https://lrrd.cipav.org.co/lrrd17/6/conr17070.htm

Dana, S. S., Rathore, B. S., & Kaul, P. N. (2000). Morbidity and mortality pattern in desi cattle reared by the Santal tribe of West Bengal. *Indian Journal of Animal Research*, *34*(1), 49–51.

Danho, T., Kacou, A., Bodjo, S. ., & Couacy-Hymannn, E. (2006). Amelioration de la production en aviculture familiale: cas de la Cote d’Ivoire. *Improving Farmyard Poultry Production in Africa: Interventions and Their Economic Assessment*. https://www-pub.iaea.org/mtcd/publications/pdf/te_1489_web.pdf

Ding, Q., Liu, W., Ni, Q., Zhang, G., & Bai, A. (2011). Diagnosis and treatment of leukocytozoonosis in chickens (EN). *Poultry Science*. https://chn.oversea.cnki.net/KCMS/detail/detail.aspx??sfield=fn&QueryID=9&CurRec=18&recid=&FileName=COOK201109036&DbName=CJFD2011&DbCode=CJFD&yx=&pr=&URLID=

Du, Y. (2017). Diagnosis and control of chicken colibacillosis (EN). *Agricultural Engineering Technology*, *37*(20). https://doi.org/10.16815/j.cnki.11-5436/s.2017.20.064

Feng, G. (1997). Diagnosis and treatment of chicken Riley’s Taeniasis (EN). *Veterinary Pharmaceuticals & Feed Additives*. https://xueshu.baidu.com/usercenter/paper/show?paperid=fc800ca22de83aa67589404880566166&site=xueshu_se

Geerlings, E. (2007). *Highly pathogenic avian influenza: a rapid assessment of its socio-economic impact on vulnerable households in Egypt* (Issue June). https://doi.org/10.13140/RG.2.1.3335.3686

Haunshi, S., Saxena, S. C., Biswajit, D., & Bujarbaruah, K. M. (2007). Comparative performance of Vanaraja chicken under backyard and intensive system at climatic conditions of Meghalaya. *Indian Journal of Animal Sciences*, *1*(99–102).

Hernandez-Divers, S. M., Villegas, P., Prieto, F., Unda, J. C., Stedman, N., Ritchie, B., Carroll, R., & Hernandez-Divers, S. J. (2006). A survey of selected avian pathogens of backyard poultry in northwestern Ecuador. *Journal of Avian Medicine and Surgery*, *20*(3), 147–158. https://doi.org/10.1647/2005-015R.1

Hien, O. C., Diarra, B., Dabire, R., Wangrawa, J., & Sawadogo, L. (2011). Effects of external parasites on the productivity of poultry in the traditional rearing system in the sub-humid zone of Burkina Faso. *International Journal of Poultry Science*, *10*(3), 189–196. https://doi.org/10.3923/ijps.2011.189.196

Hu, Y. (2008). *The prevention and control of coccidiosis in Xiang Huang chicken (EN)* [Hunan Agricultural University]. https://www.cnki.net/KCMS/detail/detail.aspx?dbcode=CMFD&dbname=CMFD2009&filename=2009090983.nh&uniplatform=OVERSEA&v=gAICFJLwtpLIBVgdnmd-xsXi9FunrrXJ5kzPvF7B3VFymrkTC9v9d1wXSaxMNuTX

Huang, M. (2020). Diagnosis and treatment of a chicken colibacillosis and coccidiosis mixed infection (EN). *Animals Breeding and Feed*. https://chn.oversea.cnki.net/KCMS/detail/detail.aspx?dbcode=CJFD&dbname=CJFDLAST2020&filename=YZCL202010041&uniplatform=OVERSEAS_CHS&v=ze-Cizx1TwvSHxWxg-osD1LEJ72JrQdNibo-jhtvRakH3cSCgFhzGoQ8fA01Uy3m

Huang, Y., & Tang, M. (2013). Diagnosis and treatment of chicken infectious bursal disease (EN). *Chinese Journal of Animal Husbandry and Veterinary Medicine*. https://wenku.baidu.com/view/3611d0b8d2f34693daef5ef7ba0d4a7303766c40.html?_wkts_=1683099920485&bdQuery=一起鸡传染性法氏囊病的诊治体会+黄艳庭%2C唐明军+%28陕西省南郑县福成镇兽医站%2C南郑

Janvier, M. (2017). *Characterization of production systems and development of breeding objectives for indigenous chicken in Rwanda* [Egerton]. http://41.89.96.81:8080/xmlui/handle/123456789/1407

Jiao, P., Chen, G., Wu, K., Yang, Y., Hou, X., Liu, T., & Wei, P. (2015). Diagnosis and treatment of secondary infection of Escherichia coli in outbreak of Marek’s disease in black-bone chicken (EN). *China Poultry*. https://chn.oversea.cnki.net/KCMS/detail/detail.aspx?dbcode=CJFD&dbname=CJFDLAST2016&filename=ZGJQ201522019&uniplatform=OVERSEA&v=bdt_RfO8sH5FO1hn_dOZzA2V4D8FeWHB2dO0kWZBl5hHRb8pajM01zo6xciv0KdM

Karo-Karo, D., Diyantoro, Pribadi, E. S., Sudirman, F. X., Kurniasih, S. W., Sukirman, Indasari, I., Muljono, D. H., Koch, G., & Stegeman, J. A. (2019). Highly pathogenic avian influenza a(H5n1) outbreaks in West Java Indonesia 2015–2016: Clinical manifestation and associated risk factors. *Microorganisms*, *7*(9). https://doi.org/10.3390/microorganisms7090327

Katoch, R., Yadav, A., Godara, R., Khajuria, J. K., Borkataki, S., & Sodhi, S. S. (2012). Prevalence and impact of gastrointestinal helminths on body weight gain in backyard chickens in subtropical and humid zone of Jammu, India. *Journal of Parasitic Diseases*, *36*(1), 49–52. https://doi.org/10.1007/s12639-011-0090-z

Khadda, B. S., Lata, K., Kumar, R., Jadav, J. K., Singh, B., & Palod, J. (2017). Production performance and economics of CARI Nirbheek chicken for backyard farming under semi-arid ecosystem in central Gujarat, India. *Indian Journal of Animal Research*, *51*(2), 382–386. https://doi.org/10.18805/ijar.8421

Khalafall, A. ., Abdel Aziz, S. ., & Elhassan, S. . (2006). Increasing family poultry production in the Sudan through Newcastle disease control and improving housing. *Improving Farmyard Poultry Production in Africa: Interventions and Their Economic Assessment*. https://www-pub.iaea.org/mtcd/publications/pdf/te_1489_web.pdf

Koko, M., Maminiaina, O. ., Ravaomanana, J., & Rakotonindrina, S. . (2006). Impacts de l’amelioration de conduire sur la productivite de l’aviculture villageoise a Madagascar. *Improving Farmyard Poultry Production in Africa: Interventions and Their Economic Assessment*. https://www-pub.iaea.org/mtcd/publications/pdf/te_1489_web.pdf

Kondombo, S. R., Nianogo, A. J., Kwakkel, R. P., Udo, H. M. Y., & Slingerland, M. (2003). Comparative Analysis of Village Chicken Production in Two Farming Systems in Burkina Faso. *Tropical Animal Health and Production*, *35*(6), 563–574. https://doi.org/10.1023/A:1027336610764

Kumaresan, A., Bujarbaruah, K. M., Pathak, K. A., Chhetri, B., Ahmed, S. K., & Haunshi, S. (2008). Analysis of a village chicken production system and performance of improved dual purpose chickens under a subtropical hill agro-ecosystem in India. *Tropical Animal Health and Production*, *40*(6), 395–402. https://doi.org/10.1007/s11250-007-9097-y

Kye, S. J., Kim, J. Y., Seul, H. J., Kim, S., Kim, S. E., Lee, H. S., Sorn, S., & Choi, K. S. (2013). Phylogenetic analysis and genetic characterization of chicken anemia virus isolates from Cambodia. *Poultry Science*, *92*(10), 2681–2686. https://doi.org/10.3382/ps.2013-03204

Li, Y. (2014). A report on the prevention and treatment of black bone chicken pullorum (EN). *Technical Advisor for Animal Husbandry*. https://chn.oversea.cnki.net/KCMS/detail/detail.aspx?dbcode=CJFD&dbname=CJFD2014&filename=HLCM201406085&uniplatform=OVERSEAS_CHS&v=a3zjNOHQ7TK1fCXqk72IR2kcKtMAouGe27pO01OY4o30TulWwPFeHNTA3MFlaMmw

Liu, Dandan, Kong, L., Tao, J., & Xu, J. (2018). An outbreak of histomoniasis in backyard sanhuang chickens. *Korean Journal of Parasitology*, *56*(6), 597–602. https://doi.org/10.3347/kjp.2018.56.6.597

Liu, Dechen. (2013). Symptoms and control of Newcastle disease secondary to chicken coccidiosis (EN). *Technical Advisor for Animal Husbandry*. https://chn.oversea.cnki.net/KCMS/detail/detail.aspx?dbcode=CJFD&dbname=CJFD2013&filename=HLCM201302120&uniplatform=OVERSEAS_CHS&v=zvTt5D5tQ1R8scggdi1LdYZ-boZ5_InBWPUQqab5KY-ez309_rR0dn_ojxfn-oiF

Maminiaina, O. F., Koko, M., Ravaomanana, J., & Rakotonindrina, S. J. (2007). Épidémiologie de la maladie de Newcastle en aviculture villageoise à Madagascar. *OIE Revue Scientifique et Technique*, *26*(3), 691–700. https://doi.org/10.20506/rst.26.3.1776

Meng, X., & Zhou, Y. (1990). Clinical measures of broiler bursitis (EN). *Jiaqin Jiyao*. https://xueshu.baidu.com/usercenter/paper/show?paperid=893ddf391afeb47604bc847ce79772c4&site=xueshu_se

Moharam, I., Razik, A. A. el, Sultan, H., Ghezlan, M., Meseko, C., Franzke, K., Harder, T., Beer, M., & Grund, C. (2019). Investigation of suspected Newcastle disease (ND) outbreaks in Egypt uncovers a high virus velogenic ND virus burden in small-scale holdings and the presence of multiple pathogens. *Avian Pathology*, *48*(5), 406–415. https://doi.org/10.1080/03079457.2019.1612852

Mourad, M., Bah, A. S., & Gbanamou, G. (1997). Evaluation de la productivité et de la mortalité de la poule locale sur le plateau du Sankaran, Faranah, Guinée, en 1993-1994. *Revue d’élevage et de Médecine Vétérinaire Des Pays Tropicaux*, *50*(4), 343–349. https://doi.org/10.19182/remvt.9566

Mwalusanya, N. A., Katule, A. M., Mutayoba, S. K., Mtambo, M. M. A., Olsen, J. E., & Minga, U. M. (2001). Productivity of local chickens under village management conditions. *Tropical Animal Health and Production*, *34*(5), 405–416. https://doi.org/10.1023/A:1020048327158

Nahimana, G., Missohou, A., Ayssiwede, S. B., Cissé, P., Butore, J., & Touré, A. (2017). Amélioration de la survie des poussins et des performances zootechniques de la poule locale en condition villageoise au Sénégal. *Revue d’élevage et de Médecine Vétérinaire Des Pays Tropicaux*, *70*(1), 3. https://doi.org/10.19182/remvt.31393

Nchinda, V. P. (2010). *Performance of Family Poultry (Chicken) Husbandry Program in Artibonite and Southern Departments of Haiti: Prospects and Way Forward* (Issue December).

Nguyen, T. B. V., Cuong, N. V., Yen, N. T. P., Nhi, N. T. H., Kiet, B. T., Hoang, N. V., Hien, V. B., Thwaites, G., Carrique-Mas, J. J., & Ribas, A. (2020). Characterisation of gastrointestinal helminths and their impact in commercial small-scale chicken flocks in the Mekong Delta of Vietnam. *Tropical Animal Health and Production*, *52*(1), 53–62. https://doi.org/10.1007/s11250-019-01982-3

Padhi, M. K., Chatterjee, R. N., & Rajkumar, U. (2014). A study on performance of a crossbred chicken developed using both exotic and indigenous breeds under backyard system of rearing. *Journal of Poultry Science and Technology*, *2*(2), 26–29.

Rahman, M., Sorensen, P., Jensen, H. A., & Dolberg, F. (1997). Exotic hens under semi scavenging conditions in Bangladesh. *Livestock Research for Rural Development*, *9*(3). http://lrrd.cipav.org.co/lrrd9/3/bang931.htm

Rodriguez, J. C., Segura, J. C., Alzina, A., & Gutierrez, M. A. (1997). Factors affecting mortality of crossbred and exotic chickens kept under backyard systems in Yucatan, Mexico. *Tropical Animal Health and Production*, *29*(3), 151–157. https://doi.org/10.1007/BF02633012

Sadef, S., Khan, M. S., Rehman, M. S., Ibrahim, M. N. M., & Okeyo, A. M. (2015). Flock composition and pattern of entry and exit of village chickens in Punjab (Pakistan). *Tropical Agricultural Research*, *26*(3), 448–455. https://doi.org/10.4038/tar.v26i3.8108

Samal, P., Panda, A. K., Boitai, S. S., & ... (2017). Caecal-coccidiosis in Vanaraja chicks and its successful therapeutic management. *Pharma …*, *6*(11), 827–828. https://www.thepharmajournal.com/archives/2017/vol6issue11/PartL/6-11-126-624.pdf

Sarma, M., Islam, R., Borah, M. K., Sharma, P., Mahanta, J. D., Kalita, N., & Bhattacharyya, B. N. (2018). Comparative performance of vanaraja, srinidhi and desi chicken under traditional system among tribal community of Assam. *Indian Journal of Animal Research*, *52*(10), 1518–1520. https://doi.org/10.18805/ijar.B-3391

Semakula, J., Lusembo, P., Mwesigwa, M., Ssennyonjo, J., Lumu, R., Kugonza, D. R., & Mutetikka, D. (2015). Infectious causes of mortality in smallholder mature scavenging chicken in Central Uganda. *Livestock Research for Rural Development*, *27*(4).

Shao, S., Li, J., Nie, Y., Mu, G., Li, G., Wang, T., & White, S. (2016). Diagnosis and treatment of mixed infection of Escherichia coli and coccidiosis in Guinea chickens raised in undergrowth (EN). *Modern Agricultural Science and Technology*. https://chn.oversea.cnki.net/KCMS/detail/detail.aspx?dbcode=CJFD&dbname=CJFDLAST2016&filename=ANHE201613180&uniplatform=OVERSEAS_CHS&v=HUUJ-FbMO2ZLOkXXKSEZJcpBI4WaYXVCwMfP_VIYzYd4ZYQfju13mBgGIHXGuNp_

Skallerup, P., Luna, L. A., Johansen, M. V., & Kyvsgaard, N. C. (2005). The impact of natural helminth infections and supplementary protein on growth performance of free-range chickens on smallholder farms in El Sauce, Nicaragua. *Preventive Veterinary Medicine*, *69*(3–4), 229–244. https://doi.org/10.1016/j.prevetmed.2005.02.003

Sun, D. (1990). Infectious supracoelomal bursal disease in 18-week-old chickens (EN). *Poultry Collection*, *4*. http://www.cqvip.com/Main/Detail.aspx?id=299008

Tang, L., Luo, Y., Jing, T., Gao, G., & Wan, G. (1986). Staphylococcal disease in chicken (EN). *Xinjiang Agricultural Sciences*. https://chn.oversea.cnki.net/KCMS/detail/detail.aspx?dbcode=CJFD&dbname=CJFD8589&filename=XJNX198605023&uniplatform=OVERSEAS_CHS&v=YKupksYqgZYxJUCbtLwZT7rkewRhrmanX78ePpLjulMQ_881QJGxyouDR8MgwicZ

Tiensin, T., Chaitaweesub, P., Songserm, T., Chaisingh, A., Hoonsuwan, W., Buranathai, C., Parakamawongsa, T., Premashthira, S., Amonsin, A., Gilbert, M., Nielen, M., & Stegeman, A. (2005). Highly Pathogenic Avian Influenza H5N1, Thailand, 2004. *Emerging Infectious Diseases*, *11*(11).

Van, N. T. B., Yen, N. T. P., Nhung, N. T., Cuong, N. Van, Kiet, B. T., Hoang, N. Van, Hien, V. B., Chansiripornchai, N., Choisy, M., Ribas, A., Campbell, J., Thwaites, G., & Carrique-Mas, J. (2020). Characterization of viral, bacterial, and parasitic causes of disease in small-scale chicken flocks in the Mekong Delta of Vietnam. *Poultry Science*, *99*(2), 783–790. https://doi.org/10.1016/j.psj.2019.10.033

Vijayalingam, T. A., Rajesh, N. V., & Ilavarasan, S. (2019). A report on intestinal coccidiosis in a Kairali Desi chicken farm in Ramanathapuram district. *Journal of Entomology and Zoology Studies*, *7*(2), 965–968.

Wang, X., & Wen, Y. H. (2012). Diagnosis and treatment of common cold and coccidiosis in free-range chickens (EN). *Journal of Traditional Chinese Veterinary Medicine*. https://chn.oversea.cnki.net/KCMS/detail/detail.aspx?dbcode=CJFD&dbname=CJFD2012&filename=ZSZZ201205020&uniplatform=OVERSEAS_CHS&v=SOgEvhkkpeup4BDzAuzpAVK8C8dsxOkCVZgGvXS4UQGGuPimdbTGbBILqkkXM51v

Wang, Y., Chen, L., Cui, T., Zhang, T., Liu, M., & Xue, L. (2016). Diagnosis and control of coccidiosis in free-range chickens raised under the forest (EN). *Zhongguo Xumu Shouyi Wenzhaishuoyi*. https://chn.oversea.cnki.net/KCMS/detail/detail.aspx??sfield=fn&QueryID=0&CurRec=1&recid=&FileName=ZXWA201603208&DbName=CJFDLAST2016&DbCode=CJFD&yx=&pr=&URLID=

Wilson, T. M., Sousa, S. K. H., Paludo, G. R., de Melo, C. B., Llano, H. A. B., Soares, R. M., & Castro, M. B. (2020). An undescribed species of Sarcocystis associated with necrotizing meningoencephalitis in naturally infected backyard chickens in the Midwest of Brazil. *Parasitology International*, *76*(February), 102098. https://doi.org/10.1016/j.parint.2020.102098

Xiong, D., Qin, C., Liu, L., & Zhang, L. (2016). Diagnosis and treatment of coccidiosis complicated by colibacillosis in free-range chickens (EN). *Poultry Science*. https://chn.oversea.cnki.net/KCMS/detail/detail.aspx?dbcode=CJFD&dbname=CJFDLAST2016&filename=COOK201601019&uniplatform=OVERSEAS_CHS&v=BqP881qPJkglYB7i6hdTQ4Z3B7N7zrmKCzrlM9EnEn8vk0ZjaZ6kNIecke65_Uz3

Xu, D., & Chen, C. (2007). Mixed infection of atypical Newcastle disease and small intestina coccidiosis in Noma chickens (EN). *Poultry Husbandry and Disease Control*. https://chn.oversea.cnki.net/KCMS/detail/detail.aspx??sfield=fn&QueryID=3&CurRec=1&recid=&FileName=YQYF200703027&DbName=CJFD2007&DbCode=CJFD&yx=&pr=&URLID=

Yang, Y. (2004). Diagnosis and treatment of Newcastle disease in chicks (EN). *Guide to Chinese Poultry*. https://chn.oversea.cnki.net/KCMS/detail/detail.aspx?dbcode=CJFD&dbname=CJFD2004&filename=QYKJ200406025&uniplatform=OVERSEAS_CHS&v=hRqPhsv195ZBSiPRJ18XsBhcHYyv-qtB7-VAHycGteA0EgewusnqYPk6cFNgKcZE

You, L., & Diao, X. (2007). Assessing the potential impact of avian influenza on poultry in West Africa: A spatial equilibrium analysis. *Journal of Agricultural Economics*, *58*(2), 348–367. https://doi.org/10.1111/j.1477-9552.2007.00099.x

Yu, F. (2014). Diagnosis of a chicken histomoniasis (EN). *Chinese Journal of Animal Husbandry and Veterinary Medicine*. https://wenku.baidu.com/view/aa7862d5cc22bcd126ff0ce9.html?_wkts_=1683100251558&bdQuery=一例鸡组织滴虫病的诊断+于伏国

Zhang, C. (2018). A report on diagnosis and treatment of acute coccidiosis in free-range native chickens raised in orchard (EN). *Hubei Journal of Animal and Veterinary Sciences*. https://chn.oversea.cnki.net/KCMS/detail/detail.aspx??sfield=fn&QueryID=0&CurRec=1&recid=&FileName=HXMY201808016&DbName=CJFDLAST2018&DbCode=CJFD&yx=&pr=&URLID=

Zhang, F., Cheng, S., & Jia, Y. (2004). Diagnosis and treatment of leukocytozoonosis in Zhuanghe big bone chickens (EN). *Animal’s Clinic*. https://chn.oversea.cnki.net/KCMS/detail/detail.aspx?dbcode=CJFD&dbname=CJFD2004&filename=LNXM200406023&uniplatform=OVERSEAS_CHS&v=hPqVyteKPSp6k8s7i1AlGkrE4JIC-vHaBs_AcSf9RsV9DRJRkc5IzirlIjylda5u

Zhang, M. (1999). Diagnosis and treatment of co-occurrence of pasteurellosis in chickens, turkeys and rabbits (EN). *Tianjing Journal of Animal Husbandry and Veterinary Medicine*. https://chn.oversea.cnki.net/KCMS/detail/detail.aspx??sfield=fn&QueryID=0&CurRec=2&recid=&FileName=TJXM901.023&DbName=CJFD9899&DbCode=CJFD&yx=&pr=&URLID=

Zhang, Z., Jiang, S., Yu, X., & Cui, Y. (1991). Diagnosis report on outbreak of chicken coccidiosis in breeding chickens (EN). *Jilin Animal Husbandry and Veterinary Medicine*. https://chn.oversea.cnki.net/KCMS/detail/detail.aspx??sfield=fn&QueryID=0&CurRec=2&recid=&FileName=JLXS199102016&DbName=CJFD9093&DbCode=CJFD&yx=&pr=&URLID=

Zuo, X. (2003). Diagnosis and treatment of streptococcosis in laying hens (EN). *Guide to Chinese Poultry*. http://vip.hnadl.cn/article/detail.aspx?id=10388386
