# Supplementary material for: Economic impact of chicken diseases and other causes of morbidity or mortality in backyard farms in low-income and middle-income countries: a systematic review and meta-analysis
Source: BMC Vet Res. 2025 Mar 7;21:151. doi: 10.1186/s12917-025-04549-7 (PMC11887245; doi:10.1186/s12917-025-04549-7)
Supplement: Supplementary file 9 — Additional file 9. Critical appraisal tool. [file 12917_2025_4549_MOESM9_ESM.docx]

# Critical appraisal tool

Additional Table 2. This critical appraisal tool is to determine eligible studies for the systematic review and meta-analyses on the impact of chicken diseases and other causes of morbidity or mortality in backyard chickens. To pass the quality assessment, all elements with a (*) should have a “Yes” as an answer.

| Domain | Elements |
| --- | --- |
| Research question | - Clearly defined and appropriate (Yes/No)* |
| Selection of the study population | - Description of study population (Yes/No)* - Specific inclusion and exclusion criteria (Yes/No)* - Appropriate method for sampling (Yes/No) |
| Exposure variable(s) | - The exposure clearly defined when assessed (Yes/No) |
| Outcome(s) | - Primary/secondary outcome(s) are clearly defined (Yes/No)* - Method to measure the outcome(s) is valid and reliable (Yes/No) |
| Statistical analysis | - Appropriate statistical analysis (Yes/No) - Assessment of confounding (Yes/No) |
| Results | - Appropriate measure of effect and precision for outcomes (Yes/No)* |
| Discussion | - Conclusions are supported by results with possible biases (Yes/No)* - Limitations are taken into consideration (Yes/No) |
| Conflict of interest | - Assessment of the declaration of conflict of interest in regard to the inclusion of bias |
