# Supplementary material for: Economic impact of chicken diseases and other causes of morbidity or mortality in backyard farms in low-income and middle-income countries: a systematic review and meta-analysis
Source: BMC Vet Res. 2025 Mar 7;21:151. doi: 10.1186/s12917-025-04549-7 (PMC11887245; doi:10.1186/s12917-025-04549-7)
Supplement: Supplementary file 10 — Additional file 10. Assessment of risk of bias. [file 12917_2025_4549_MOESM10_ESM.docx]

# Assessment of risk of bias

Additional table 3. The assessment of risk of bias was conducted in four elements of selected studies for the systematic review and meta-analyses on the impact of chicken diseases and other causes of morbidity or mortality in backyard chickens. Each element was assessed as S=strong, W= weak and U=unclear.

| Number | Study | Study population^1^ | Outcome definition^2^ | Outcome measurement^3^ | Statistics appropriate^4^ |
| --- | --- | --- | --- | --- | --- |
| #1 | Skallerup et al., 2005 | S | S | S | S |
| #2 | Annand et al., 2021 | S | S | S | S |
| #3 | Biswas et al., 2008 | S | S | S | S |
| #4 | Hernandez-Divers et al., 2006 | S | S | S | S |
| #5 | Haunshi et al., 2007 | S | S | S | S |
| #6 | Katoch et al., 2012 | S | S | S | S |
| #7 | Kondombo et al., 2003 | W | W | W | S |
| #8 | Liu et al., 2018 | S | S | S | W |
| #9 | Biswas et al., 2006 | S | S | S | W |
| #10 | Kumaresan et al., 2008 | S | S | W | S |
| #11 | Padhi et al., 2014 | S | S | W | S |
| #12 | Alfred et al., 2012 | S | S | W | W |
| #13 | Hien et al., 2011 | S | S | W | S |
| #14 | Semakula et al., 2015 | S | S | S | S |
| #15 | Sarma et al., 2018 | S | S | W | S |
| #16 | Banja et al., 2017 | S | S | W | W |
| #17 | Conroy et al., 2005 | S | W | W | W |
| #18 | Khadda et al., 2017 | S | S | W | S |
| #19 | Karo-Karo et al., 2019 | S | S | W | S |
| #20 | Barman et al., 2010 | S | S | W | S |
| #21 | Samal et al., 2017 | S | S | S | U |
| #22 | Akanbi et al., 2014 | S | S | W | U |
| #23 | Wilson et al., 2020 | S | S | W | U |
| #24 | Rahman et al., 1997 | S | S | S | S |
| #25 | Nchinda, 2010 | S | S | S | S |
| #26 | Janvier, 2017 | S | S | W | W |
| #27 | Choe-ngern et al., 1993 | S | S | W | U |
| #28 | Mourad et al., 1997 | S | S | S | S |
| #29 | Sadef et al., 2015 | S | S | W | W |
| #30 | Nahimana et al., 2017 | S | S | W | S |
| #31 | Mwalusanya et al., 2001 | S | S | W | S |
| #32 | Azzam et al., 2006 | S | S | S | S |
| #33 | Danho et al., 2006 | S | S | W | S |
| #34 | Koko et al., 2006 | S | S | W | S |
| #35 | Khalafall et al., 2006 | S | S | S | W |
| #36 | Bessell et al., 2020 | S | S | W | S |
| #37 | Nguyen et al., 2020 | S | S | S | S |
| #38 | Tiensin et al., 2005 | S | S | S | S |
| #39 | Geerlings, 2007 | S | S | W | W |
| #40 | Dana et al., 2000 | S | S | W | W |
| #41 | Du, 2017 | S | S | S | W |
| #42 | Hu, 2008 | W | S | S | S |
| #43 | Feng, 1997 | S | S | S | S |
| #44 | Ding et al., 2011 | S | S | W | S |
| #45 | Xu et al., 2007 | S | S | S | S |
| #46 | Zhang et al., 2004 | S | S | S | S |
| #47 | Zhang et al., 1999 | S | S | S | S |
| #48 | Zhang et al., 1991 | S | S | S | S |
| #49 | Chen, 1992 | S | S | S | S |
| #50 | Huang, 2020 | S | S | S | S |
| #51 | Zhang, 2018 | S | S | S | S |
| #52 | Shao et al., 2016 | S | S | S | S |
| #53 | Wang et al., 2016 | S | S | S | S |
| #54 | Xiong et al., 2016 | S | S | S | S |
| #55 | Jiao et al., 2015 | S | S | S | S |
| #56 | Li, 2014 | S | S | S | S |
| #57 | Huang et al., 2013 | S | S | S | S |
| #58 | Yu, 2014 | S | S | S | S |
| #59 | Tang et al., 1986 | S | S | S | S |
| #60 | Wang et al., 2012 | S | S | S | S |
| #61 | Liu, 2013 | S | S | S | S |
| #62 | Yang, 2004 | S | S | S | S |
| #63 | Zuo, 2003 | S | S | S | S |
| #64 | Meng et al., 1990 | S | S | S | S |
| #65 | Sun, 1990 | S | S | S | S |
| #66 | Rodriguez et al., 1997 | S | S | S | S |
| #67 | Biswas et al., 2005 | S | S | S | S |
| #68 | Bell et al., 1995 | S | S | W | W |
| #69 | Barua et al., 2006 | S | S | S | U |
| #70 | Maminiaina et al., 2007 | S | S | S | S |
| #71 | Moharam et al., 2019 | S | S | S | U |
| #72 | Vijayalingam et al., 2019 | S | S | S | U |
| #73 | Assefa et al., 2016 | S | S | W | S |
| #74 | Van et al., 2020 | S | S | S | S |
| #75 | Kye et al., 2013 | S | S | S | U |
| #76 | You et al., 2007 | S | S | S | S |
| #77 | You et al., 2007 | S | S | S | S |
| #78 | Bhuiyan et al., 2004 | S | S | S | S |

^1^: Appropriate selection of the study population and sampling methods; ^2^: Clearly definition of outcome measure; ^3^: Valid and standard measurement method (s); ^4^: Appropriate statistics method(s) for the analysis.
