# Supplementary material for: Economic impact of chicken diseases and other causes of morbidity or mortality in backyard farms in low-income and middle-income countries: a systematic review and meta-analysis
Source: BMC Vet Res. 2025 Mar 7;21:151. doi: 10.1186/s12917-025-04549-7 (PMC11887245; doi:10.1186/s12917-025-04549-7)
Supplement: Supplementary file 11 — Additional file 11. Results of the Peter regression tests. [file 12917_2025_4549_MOESM11_ESM.docx]

# Results of the Peter regression tests

Additional table 4. Results of the Peter regression tests to assess for asymmetry.

| Mortality cause | Number of estimates (k) | T-value | P-value* | Interpretation |
| --- | --- | --- | --- | --- |
| Predation | 23 | 0.71 | 0.4860 | No funnel plot asymmetry |
| Injuries | 7 | - | - | Test cannot be performed because k<10 |
| Cachexia | 2 | - | - | Test cannot be performed because k<10 |
| Weather | 2 | - | - | Test cannot be performed because k<10 |
| Fungus | 2 | - | - | Test cannot be performed because k<10 |
| Bacterial diseases | 15 | 0.95 | 0.3590 | No funnel plot asymmetry |
| Bacteria and parasitic diseases | 3 | - | - | Test cannot be performed because k<10 |
| Bacterial and viral diseases | 16 | 1.29 | 0.7634 | No funnel plot asymmetry |
| Parasitic diseases | 21 | 0.33 | 0.7432 | No funnel plot asymmetry |
| Viral diseases | 33 | 0.91 | 0.3699 | No funnel plot asymmetry |

**p-value<0.05 considered significant*
